# Precursor-Directed Biosynthesis of Aminofulvenes: New Chalanilines from Endophytic Fungus *Chalara* sp.

**DOI:** 10.3390/molecules26154418

**Published:** 2021-07-22

**Authors:** Mahsa Khoshbakht, Jason Srey, Donovon A. Adpressa, Annika Jagels, Sandra Loesgen

**Affiliations:** 1 Department of Chemistry, Oregon State University, Corvallis, OR 97331, USA; khoshbam@oregonstate.edu (M.K.); sreyj@oregonstate.edu (J.S.); donovon.adpressa@gmail.com (D.A.A.); 2 Whitney Laboratory for Marine Bioscience, Department of Chemistry, University of Florida, Gainesville, FL 32080, USA; annika.jagels@whitney.ufl.edu

**Keywords:** precursor-directed biosynthesis, fungal metabolite, aminofulvenes, biotransformation

## Abstract

The plant endophyte *Chalara* sp. is able to biotransform the epigenetic modifier vorinostat to form unique, aniline-containing polyketides named chalanilines. Here, we sought to expand the chemical diversity of chalaniline A-type molecules by changing the aniline moiety in the precursor vorinostat. In total, twenty-three different vorinostat analogs were prepared via two-step synthesis, and nineteen were incorporated by the fungus into polyketides. The highest yielding substrates were selected for large-scale precursor-directed biosynthesis and five novel compounds, including two fluorinated chalanilines, were isolated, purified, and structurally characterized. Structure elucidation relied on 1D and 2D NMR techniques and was supported by low- and high-resolution mass spectrometry. All compounds were tested for their bioactivity but were not active in antimicrobial or cell viability assays. Aminofulvene-containing natural products are rare, and this high-yielding, precursor-directed process allows for the diversification of this class of compounds.

## 1. Introduction

**Figure 1:**
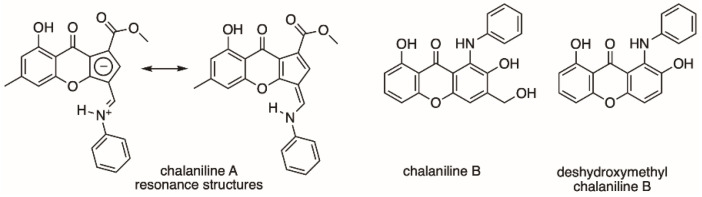
Chalanilines A and B isolated from *Chalara* sp. and deshydroxymethyl chalaniline B made by chemical synthesis.

Fungal natural products keep surprising us with unprecedented chemical diversity derived from complex biosynthetic machineries. A multitude of applications for fungal metabolites have been found to aid humankind: from the antibiotic penicillin, the cholesterol-lowering agent lovastatin, to the immunosuppressant cyclosporin, to name a few [1,2]. In recent years, an increasing number of fungal genomes have been sequenced and the number of biosynthetic gene clusters present in these fungi, which encode for specialized small molecules, is much larger than the chemical diversity found [3,4]. Together with the estimate that only a small fraction of fungi have been chemically explored thus far, the potential of chemical discovery from fungi remains high [5,6,7]. Additionally, fungi are masters in biotransformation and have been shown to harbor unique enzymes enabling them to conquer challenging environments, food sources, and lifestyles [8,9]. This has been exploited in biotechnological applications; approximately half of the commercially available enzymes used in industry are of fungal origin [10]. Some fungi are known to biotransform small molecules and/or biosynthesize highly chemoreactive species. One example is the compound maximiscin from *Tolypocladium* sp. which results from highly reactive intermediates that can detoxify various synthetic and naturally derived antifungals via nucleophilic substitutions [11]. Previously, we found that the endophyte *Chalara* sp. is able to biotransform the epigenetic modifier vorinostat, also known as suberanilohydroxamic acid, to form unique, aniline-containing polyketides named chalanilines A and B (Figure 1) [12]. In our studies, chalaniline B, an unusual aminoxanthone, exhibited micromolar antimicrobial activity against multidrug-resistant *Staphylococcus aureus* strain ATCC# BAA-44. Recently, the total synthesis of chalaniline B was achieved, which allowed us to expand its antimicrobial assessment [13]. To our surprise, the synthetic intermediate deshydroxymethyl chalaniline B (1-anilino-2,8-dihydroxyxanthone) (Figure 1) was the most potent compound tested, with MIC values of 8 μg/mL (25 μM) against both methicillin-resistant *S. aureus* and *B. subtilis*. Aniline moieties are rare in natural products [14,15], but have been used extensively by synthetic chemists, for example, in dyes and early antibiotic development, or in medicinal chemistry to optimize a drug lead [16]. In one example, the introduction of aniline substituents enhanced the antifungal activity of aminoquinolones [17,18] and in another, methoxy substituents on the aniline moiety increased brain permeability in the development of a new drug to treat Alzheimer’s disease [19]. Here, we sought to expand the chemical diversity of chalaniline A-type molecules by changing the aniline moiety in the precursor vorinostat. Precursor-directed biosynthesis yielded five novel compounds and their bioactivity in antimicrobial and cell viability assays was tested. 

## 2. Results

**Figure 2:**
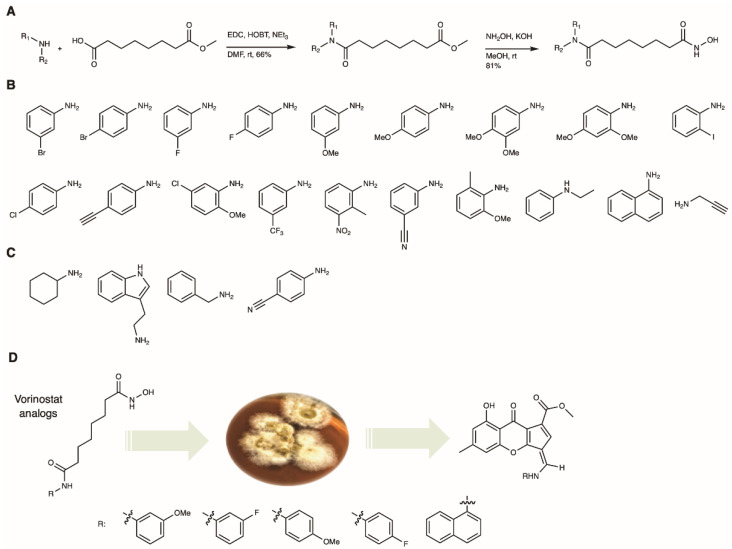
(**A**) Synthesis of twenty-three vorinostat analogs following published procedures (R_1_ and R_2_ for different amine substituents) [20]. (**B**) Nineteen amines were biotransformed into chalaniline A structures. (**C**) Four amine moieties were not incorporated by the fungus. (**D**) Overview of precursor-directed biosynthesis of chalaniline A-type molecules by supplementing *Chalara* sp. cultures with vorinostat analogs.

Vorinostat analogs were prepared in a two-step synthesis following previous reports (Figure 2A) [20]. Briefly, commercially available suberic acid monomethyl ester and different anilines were coupled using 1-(3-dimethylaminopropyl)-3-ethylcarbodiimide (EDC) and hydroxybenzotriazole (HOBt), followed by hydroxyl amination under basic conditions as the second step. After adjusting to pH 7 with hydrochloric acid, the vorinostat analogs were purified using silica column chromatography and analyzed by mass spectrometry (MS) and nuclear magnetic resonance (NMR) and the data compared to published reports [21,22,23]. Overall yields of the derivatives were 60–75% over two steps.

Next, the vorinostat analogs were supplemented to *Chalara* cultures (Figure 2D). Previously, we have found that the full vorinostat structure is required for high yields in the biotransformation; aniline or acetanilide were not incorporated or were only incorporated in trace amounts [12]. One can hypothesize that the suberoyl chain aids in cell permeability, and once inside the fungal cells, the aniline moiety is released by catabolic processes similar to the ones found in human serum [24]. Isofusidienols, as well as chalanilines from *Chalara* sp., can be envisioned biosynthetically from a xanthone arene epoxidation, forming a highly reactive aldehyde on the xanthofulvene, which is able to react with free aniline to form chalaniline A (SI Figure S33) [12]. Here, we optimized the feeding experiment and found that the addition of vorinostat precursors (in DMSO, final concentration 1 mM) to fungal cultures, followed by cultivation for 12–19 days, provided the highest yields of chalanilines in the *Chalara* cultures (SI Figures S34 and S35). In total, twenty-three different vorinostat analogs were prepared and nineteen were incorporated into the polyketide backbone by the fungus as shown by low-resolution mass spectrometry (SI Figure S36). We selected five vorinostat analogs with the most promising yields for large-scale precursor-directed biosynthesis, using 2 L cultures of fungus, and were able to isolate unoptimized yields of 1–4 mg/L of compounds **1**–**5**. 

### 2.1. Structure Elucidation 

**Figure 3:**
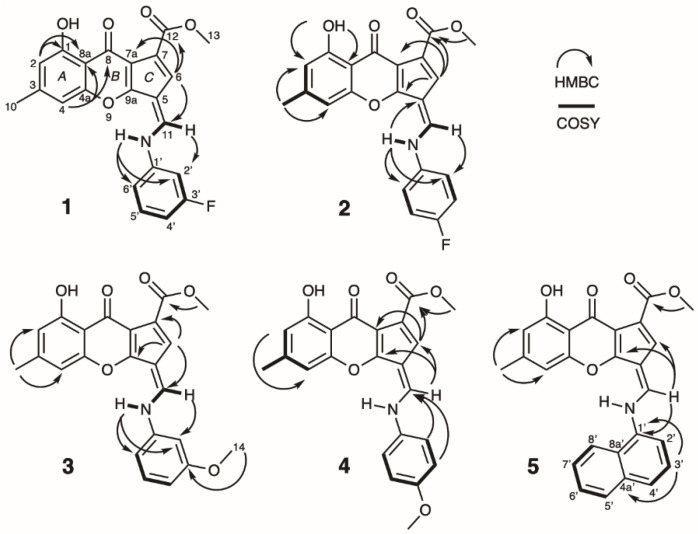
Chemical structures of new chalanilines **1**–**5** with selected 2D NMR correlations.

3-Fluoro chalaniline A (**1**) was isolated as a yellow amorphous solid in a yield of 3.2 mg/L. The HRESIMS gave an *m*/*z* value of 394.1088 [M + H]^+^ (calcd for C_22_H_17_FNO_5_, 394.1085; Δppm = 0.8) and an *m*/*z* value of 416.0904 [M + Na]^+^ (calcd for C_22_H_16_FNO_5_Na, 416.0905; Δppm = 0.2), resulting in a molecular formula of C_22_H_16_FNO_5_. The UV spectrum showed maxima at 380, 308, and 244 nm, representing the chalaniline A-type backbone. The ^1^H NMR spectrum exhibited a broad hydroxyl peak (*δ*_H_ 13.75), an N-H resonance (*δ*_H_ 11.84) with a large coupling constant (14.5 Hz) to one methine (*δ*_H_ 8.74), seven aromatic/olefinic hydrogens, one methoxy, and one methyl group (Table 1). The ^13^C NMR spectrum displayed two carbonyls, two methyl carbons, and eighteen olefinic carbons. The 3-fluoro benzene moiety exhibited ^1^*J* (244.9 Hz), ^2^*J* (26.2 and 21.4 Hz), and ^3^*J* (9.4 Hz) C-F coupling constants in the carbon spectrum, and the proton spectrum revealed two ^3^*J* (10.7 and 8.0 Hz) H-F coupling constants (Table 1). The structure of **1** was determined by conventional 2D NMR experiments. COSY and HSQC correlations helped to establish aromatic rings A to C. HMBC correlations between H-6 of the fulvene and C-11 of the bridging methine, connections between N-H and the aniline ring (C-2′ and C-6′), as well as the correlation between H-11 and C-2′ supported the structural assignment. The placement of the fluorine at C-3′ was confirmed by C-F coupling constant analysis (Figure 3).

As previously established by quantum mechanical calculations [12], chalanilines exist as zwitterions in solution (Figure 1). The NMR data found for **1–3** with the large *trans* coupling constant between N-H and H-11 confirm the iminium with delocalized double bond character.

4-Fluoro chalaniline A (**2**) was isolated as a yellow amorphous solid and the HRESIMS provided an *m*/*z* value of 394.1088 [M + H] ^+^ (calcd for C_22_H_17_FNO_5_, 394.1085; Δppm = 0.8) and *m*/*z* value of 416.0909 [M + Na]^+^ for the sodium adduct (calcd for C_22_H_16_FNO_5_Na, 416.0905; Δppm = 1.0). The ^1^H NMR spectrum of **2** was very similar to the spectrum of **1**, the only differences can be found in the number of aromatic signals and the coupling and integration pattern for the fluoro-benzene moiety. Two sets of aromatic signals, with each an integration of ~2H, supported the symmetric, *para*-substituted fluoro-aniline incorporation (Table 1). The ^13^C spectrum showed twenty signals: one methyl, one methoxy, two carbonyl, one phenolic, and fifteen olefinic carbons. Only two aromatic protons and carbons could be found due to the symmetry in the 4′-fluoro aniline moiety. ^1^*J* C-F coupling could be found for C-4′ (244.8 Hz), ^2^*J* C-F coupling of 23.0 Hz for C-3′/5′, ^3^*J* C-F coupling of 8.3 Hz for C 2′/6′, and ^4^*J* C-F coupling (2.6 Hz) for C-1′ (Table 1). Two-dimensional NMR experiments were analyzed to complete the structure suggestion for **2**, with key HMBC correlations from N-H to both the fulvene (C-5) and the 4-fluoro anilino ring C-2′/6′, and also from the H-11 to C-2′ (Figure 3).

3-Methoxy chalaniline A (**3**) was isolated as an amorphous yellow solid. The HRESIMS gave an *m*/*z* value of 406.1284 [M + H] ^+^ (calcd for C_23_H_20_NO_6_, 406.1285) and *m*/*z* value of 428.1102 [M + Na]^+^ for the sodium adduct (calcd for C_23_H_19_NO_6_Na, 428.1105; Δppm = 0.7). The ^1^H NMR spectrum of **3** showed a similar pattern of signals as previously reported for chalaniline A [12], with the addition of a second methoxy singlet (*δ*_H_ 3.84, *δ*_C_ 55.5) and fewer aromatic resonances. The ^13^C NMR spectrum exhibited twenty-three signals: three methyl peaks, two carbonyl shifts, a phenol and seventeen olefinic signals (Table 1). The methoxy group was placed *meta* to the aniline based on the observed coupling constant (Table 1). HMBC correlations between the fulvene (H-6) and the bridging methine (H-11), H-11 and the aniline ring (C-2′), as well as correlations from the N-H into the aromatic ring (C-2′; C-6′) established connectivity of the spin systems. The methoxy group exhibited a strong HMBC correlation to C-3′, confirming its *meta* position (Figure 3). 

4-Methoxy chalaniline A (**4**) was isolated as a yellow amorphous solid with low yields. The HRESIMS showed an *m*/*z* of 406.1286 [M + H] ^+^ (calcd for C_23_H_20_NO_6_, 406.1285; Δppm = 0.2), indicative of a regioisomer of **3**. The *para*-substitution in the 4-methoxy aniline moiety became evident in the proton NMR with two doublets each with an integration of ~2H (Table 1). The ^13^C signals were mainly derived from 2D spectra and were similar to the spectra of **2** and **3**. HMBC correlations from both the H-2′ and H-3′ of the aniline ring to the bridging methine (C-11), as well as the correlation between bridging methine (H-11) to the fulvene (C-6, C-9a) assisted in the structure assembly (Figure 3). 

Notably, there are differences in the NMR data between compounds **1**–**3** and **4**, **5** in the ^1^H NMR spectrum due to different isolation procedures. When acid was used, the compounds lacked the N-H signal as well as the large coupling to H-11. We believe this is due to protonation effects by water found in the NMR solvent. 

Naphthyl chalaniline A (**5**) was isolated as a yellow amorphous solid and the HRESIMS gave an *m*/*z* value of 426.1341 [M + H]^+^ (calcd for C_26_H_20_NO_5_, 426.1336; Δppm = 1.2), and an *m*/z value of 448.1160 [M + Na]^+^ for the sodium adduct (calcd for C_26_H_19_NO_5_Na, 448.1155; Δppm = 1.1) supporting a molecular formula of C_26_H_19_NO_5_. The ^1^H and ^13^C NMR spectra were similar to chalaniline A with additional signals for the naphthyl moiety instead of the phenyl in the aromatic region (Table 1). Due to the low amount of **5**, only a partial assignment of the naphthyl moiety based on 1D NMR and chemical shift predictions, but not 2D correlations, could be made (Table 1). HMBC correlations from the bridging methine (H-11) to the fulvene (C-6, C-9a) and C-2′ of the naphthyl ring were utilized to connect the spin systems (Figure 3).

### 2.2. Bioactivity 

Inhibition of cell viability of pure compounds **1**-**5** was evaluated against colon (HCT-116) and melanoma (SK-MEL-5) cancer cell models by measuring the reduction of the tetrazolium salt MTT (3-(4, 5 dimethylthiazolyl-2)-2,5-diphenyltetrazolium bromide) by metabolically active cells [25]. No inhibition of cell viability was observed at a single dose of 10 μM against these cell lines. All isolated compounds displayed no antimicrobial activity at a dose of 128 μg/mL against a panel of four Gram-positive bacteria (*S. aureus* ATCC 25923, methicillin-resistant *S. aureus* ATCC BAA-41, multidrug-resistant *S. aureus* ATCC BAA-44, and *E. faecium* ATCC 49032), two Gram-negative bacteria (*P. aeruginosa* ATCC 15422, *E. coli* ATCC 8739) as well as the yeast *Candida albicans* (ATCC 90027) following CLSI guidelines (details in the experimental section) [26]. 

## 3. Discussion

Precursor-directed biosynthesis has emerged as a powerful tool to diversify complex natural products [27]. It combines the best of two worlds; the flexibility and ease of organic synthesis to access small molecule precursors with the selectivity and often high yields found in biosynthetic processes. This strategy allows natural products to be made with “unnatural” functional groups, for example fluorine atoms or alkyne sidechains, which enable bio-orthogonal approaches for mode-of-action studies. Anilino-fulvenes are rarely found in chemical databases and chalaniline A was the first example found in fungi to our knowledge. The ability to utilize the fungal biosynthesis to incorporate different anilino moieties enabled us to study this new compound class in more detail. *Chalara* sp. was able to biotransform nineteen different vorinostat analogs; in particular, various anilines were well tolerated. The incorporation of alkynes or halogens like fluorine and bromine into a small molecule backbone can assist in the pull down of a potential molecular target via click-chemistry, fluorine-19 NMR to enable binding studies to macromolecules, or bromination, which might aid in crystallization attempts or ease in mass spectrometric detection [28]. These modifications also provide reactive handles for further chemical diversification efforts. We will continue our exploration of the activity of the chalanilines in an in vivo zebrafish behavior assay [29] as well as in silico molecular docking to find potential receptors and intracellular targets for these aminofulvenes.

## 4. Materials and Methods

General experimental procedures. UV spectra were recorded on a BioRad SmartSpec3000. IR spectra were recorded on a Thermo Scientific Nicolet 6700 FT-IR spectrometer (Thermo Fisher Scientific, Waltham, MA, USA). NMR spectra were acquired on a Bruker Avance III 500 MHz or Bruker Avance III 700 MHz spectrometer equipped with a 5 mm TXI probe or 5 mm BBO probe (Bruker, Billerica, MA, USA 500 MHz and 700 MHz). For compound 4, we used AMRIS’ Agilent VNMRS-600 spectrometer with a unique 1.5 mm High Temperature Superconducting (HTS) Cold Probe and AMRIS’ Bruker Neo-600 spectrometer equipped with a 1.7 mm TCI Cryoprobe, all with the residual solvent used as an internal standard (DMSO: 2.50/39.50 ppm). Low-resolution ESI-MS and HRTOFMS mass spectra were recorded in positive ionization mode on an Agilent 1100 series LC with MSD 1946 or Agilent 1260 Infinity II LC with 6545 QTOF MS, respectively. Analytical high-pressure liquid chromatography (HPLC, Agilent, Santa Clara, CA, USA) was performed using an Agilent 1100 HPLC system equipped with a photodiode array detector. The mobile phase consisted of ultra-pure water (A) and acetonitrile (MeCN) (B), both with 0.05% formic acid. A gradient method from 10% A to 100% B in 35 min at a flow rate of 0.8 mL/min was used. The column (Phenomenex Kinetex® C18, 5 μm particle size, 150 mm × 4.6 mm, Phenomenex, Torrance, CA, USA) was re-equilibrated before each injection and the column compartment was maintained at 30 °C throughout each run. Semi-preparative HPLC (Phenomenex Kinetex^®^ C 18, 5 μm particle size, 150 mm × 10 mm) utilized isocratic elution conditions or a gradient system with a flow rate of 4 mL/min on an Agilent 1100 HPLC system operating at room temperature equipped with a photodiode array detector. Preparative HPLC (Phenomenex Luna C18, 5 μm particle size, 250 mm × 21 mm) was conducted at room temperature, using isocratic elution conditions or a gradient system with a flow rate of 20 mL/min utilizing an Agilent 1260 Infinity series HPLC equipped with a DAD detector. All samples were filtered through a 0.45 μm nylon filter or centrifuged at 14,000 rpm for five minutes before LCMS and HPLC analysis. Analytical thin layer chromatography (TLC) was performed on pre-coated silica gel 60 F254 plates (Eppendorf, Hamburg, Germany). TLC plates were visualized by UV (254 and 360 nm), and by spraying with anisaldehyde solution followed by heating at 80 °C. General reagents were from Sigma-Aldrich Corp. and VWR International.

Preparation of vorinostat analogs. A solution of suberic acid monomethyl ester (1.0 g, 5.3 mmol) in anhydrous dimethyl formamide (DMF) (20.0 mL) was treated with EDC (1.2 g, 7.7 mmol), HOBt (1.0 g, 7.3 mmol), triethylamine (2.2 mL, 15.9 mmol), and stirred at room temperature (rt) for 30 minutes. Commercially available amine derivatives were first dissolved in DMF (10.0 mL) and then added to the reaction mixture. The resulting mixtures were stirred overnight (15–20 h) at rt and then diluted with ethyl acetate (EtOAc) (30.0 mL) and washed with concentrated hydrochloric acid (2 M, 8.0 mL), water (12.0 mL), and saturated sodium chloride solution (brine, 5 mL). The organic layer was dried with sodium sulfate (Na_2_SO_4_), filtered and evaporated to afford the ester derivatives. The crude products were pure enough to directly move to the second step. The ester derivatives in methanol (MeOH) (~20.0 mL) were added to freshly prepared hydroxylamine (~20.0 mL) and potassium hydroxide (KOH) and stirred for 2 h. The solvent evaporated and the reaction mixtures were extracted with EtOAc, dried with Na_2_SO_4_ and *in vacuo*. The crude products containing vorinostat derivatives were purified with silica column chromatography and structures proved via NMR and LCMS methods.

Precursors directed biosynthesis. Cultures of *Chalara* sp. 6661 were grown for seven days in YPD medium (yeast extract 20 g/L, bacto peptone 20 g/L; dextrose 40 g/L; pH 6.0) agar at 25 °C with a day/night cycle [12,30]. Then, 1 cm^2^ sections of agar/hyphae were excised and used to inoculate 50 mL cultures of YPD. After 48 h of growth at ambient temperatures with 200 rpm shaking, 1 M solutions of synthesized vorinostat derivatives [21,22,23] in dimethyl sulfoxide (DMSO) were used to bring duplicate cultures to a final treatment concentration of 1 mM. Cultures were maintained at ambient light and temperature with 200 rpm shaking for 30 days before extraction. Two-liter cultures treated with vorinostat derivatives were prepared in a similar fashion, with 1 cm^2^ hyphae/100 mL used for inoculation and vorinostat application occurring at 48 h thereafter. Biosynthesis of chalaniline A was monitored by LCMS from the day of inoculation until 26 days of cultivation; chalaniline A production was observed after 12 days (see SI Figures S33 and S34). Precursor-directed biosynthesis cultures were allowed to grow for ~20 days before extraction, and isolation of compounds of interest.

Extraction and isolation. Fungal cultures were treated with XAD-7 resin (10% *w*/*v*) and left overnight after separation of fungal mycelia from culture broth by filtration. XAD-7 resin was collected by filtration, washed with 2 L of deionized water, and then extracted with 2 L of 1:1 acetone:methanol mixture. Extract was concentrated before partitioning between EtOAc and water. The aqueous layer was washed three times with EtOAc and organic layers were combined to be concentrated to dryness in vacuo. Crude organic extracts were first separated into fractions by normal phase chromatographic separation on an ISCO flash chromatography system (3-fluoro vorinostat-treated culture: four fractions eluted with gradient of dichloromethane (DCM):MeOH, 4-fluoro vorinostat-treated culture: five fractions eluted with a gradient of DCM:MeOH, 3-methoxy vorinostat-treated culture: six fractions eluted first with a gradient of hexane:EtOAc and then with EtOAc:MeOH, 4-methoxy vorinostat-treated culture: five fractions eluted with DCM:MeOH gradient, naphthyl vorinostat-treated culture: seven fractions eluted with first 3:1 to 0:1 gradient of hexane:EtOAc and then EtOAc:MeOH gradient) and were further isolated directly from these fractions using preparative HPLC with isocratic mobile phases (3.2 mg of **1** from fraction two using a 60%:40% MeCN:H_2_O isocratic run, 2.9 mg of **2** from fraction two by preparative HPLC using 60%:40% MeCN:H_2_O isocratic run, 3.5 mg of **3** from fraction three by using 55%:45% MeCN:H_2_O isocratic run, 1.0 mg of **4** from fraction two by using 55%:45% MeCN:H_2_O isocratic run, 1.1 mg of **5** from fraction six using 65%:35% MeCN:H_2_O isocratic run). Precursor incorporation studies were checked with LCMS analysis by preparing 10 mg/mL samples in MeCN. 

3-Fluoro chalaniline A (**1**): yellow amorphous solid; IR (ATR): ν_max_ = 3370, 2925, 2854, 1712, 1651, 1591, 1470, 1297, 1208, 1147, 827, 732 cm^−1^; UV (MeCN) λ_max_: 380, 306, 244 nm; ^13^C NMR (176 MHz, DMSO-*d*_6_) and ^1^H NMR (500 MHz, DMSO-*d_6_*) see Table 1; HRESIMS: *m*/*z* 394.1088 [M + H]^+^ (calcd for C_22_H_17_FNO_5_ 394.1085; Δppm 0.8), *m*/*z* 416.0904 [M + Na]^+^ (calcd for C_22_H_16_FNO_5_Na 416.0905; Δppm 0.2)

4-Fluoro chalaniline A (**2**): yellow amorphous solid; IR (ATR): ν_max_ = 3430, 2925, 2853, 1712, 1651, 1589, 1508, 1466, 1365, 1209, 1098 cm^−1^; UV (MeCN) λ_max_: 380, 308, 244 nm; ^13^C NMR (176 MHz, DMSO-*d*_6_) and ^1^H NMR (700 MHz, DMSO-*d*_6_) see Table 1; HRESIMS: *m*/*z* 394.1088 [M + H]^+^ (calcd for C_22_H_17_FNO_5_ 394.1085; Δppm = 0.8), *m*/*z* 416.0909 [M + Na]^+^, (calcd for C_22_H_16_FNO_5_Na 416.0905; Δppm = 1.0)

3-Methoxy chalaniline A (**3**): yellow amorphous solid; IR (ATR): ν_max_ = 3290, 2924, 2850, 1703, 1647, 1601, 1510, 1470, 1252, 1190, 1050, 840, 735 cm^−1^; UV (MeCN) λ_max_ = 383, 310, 246 nm; ^13^C NMR (176 MHz, DMSO-*d*_6_) and ^1^H NMR (700 MHz, DMSO-*d*_6_) see Table 1; HRESIMS: *m*/*z* 406.1284 [M + H]^+^ (calcd for C_23_H_20_NO_6_ 406.1285; Δppm 0.2), *m*/*z* 428.1102 [M + Na]^+^ (calcd for C_23_H_19_NO_6_Na 428.1005; Δppm 0.7) 

4-Methoxy chalaniline A (**4**): yellow amorphous solid; IR (ATR): ν_max_ = 3290, 2924, 1703, 1647, 1510, 1490, 1252, 1050, 820, 745 cm^−1^; UV (MeCN) λ_max_: 382, 308, 244 nm; ^13^C NMR (150 MHz, DMSO-*d*_6_) and ^1^H NMR (600 MHz, DMSO-*d*_6_) see Table 1; HRESIMS: *m*/*z* 406.1286 [M + H]^+^, (calcd for C_23_H_20_NO_6_ 406.1285; Δppm 0.2)

Naphthyl chalaniline A (**5**): yellow amorphous solid; IR (ATR): ν_max_ = 3410, 2926, 2852, 1737, 1647, 1614, 1465,1372, 1307, 1207, 1098, 830, 767 cm^−1^; UV (MeCN) λ_max_: 374, 305, 242, 214 nm; ^13^C NMR (176 MHz, DMSO-*d*_6_) and ^1^H NMR (500 MHz, DMSO-*d*_6_) see Table 1; HRESIMS: *m*/*z* 426.1341 [M + H]^+^ (calcd for C_26_H_20_NO_5_ 426.1336; Δppm 1.2), *m*/*z* 448.1160 [M + Na]^+^ (calcd for C_26_H_19_NO_5_Na 448.1155; Δppm 1.1)

Antimicrobial Assays. Extracts and fractions were tested for inhibitory activity against *Staphylococcus aureus* (ATCC 25923), methicillin-resistant *Staphylococcus aureus* (ATCC BAA-41), multidrug-resistant *Staphylococcus aureus* (ATCC BAA-44), *Pseudomonas aeruginosa* (ATCC 15442), *Candida albicans* (ATCC 90027), *Candida krusei* (ATCC 34135), and *Mycobacterium smegmatis* (ATCC 14468) in microbroth assays performed following an established protocol [26,31]. Fractions and pure compounds were tested at a concentration of 128 μg/mL. All human pathogens used in the study were acquired from the American Type Culture Collection (ATCC, Manassas, VA, USA).

Cell proliferation assay. Cytotoxic activities of extracts and pure compounds were evaluated against colon (HCT-116) and melanoma (SK-MEL-5) cancer models by measuring the reduction of the tetrazolium salt MTT (3-(4, 5-dimethylthiazolyl-2)-2, 5-diphenyltetrazolium bromide) by metabolically active cells following standard procedures [32,33].

## Figures and Tables

**Table 1:** ^1^H and ^13^C NMR spectroscopic data for compounds **1**–**5** (all recorded in DMSO-*d*_6_), ^a^ *J*_HH_ in Hz, ^b^ *J*_CF_, ^c^ *J*_HF_, * partially assigned from 1D data.

	1		2		3		4		5	
Pos.	δ_C_ Type	δ_H_	δ_C_ Type	δ_H_	δ_C_ Type	δ_H_	δ_C_ Type	δ_H_	δ_C_ Type	δ_H_
1	161.3, C		161.8, C		161.3, C		161.9, C		161.7, C	
2	111.2, CH	6.57, s	111.5, CH	6.56, s	111.0, CH	6.56, s	109.3, CH	6.37, s	109.8, CH	6.41, s
3	145.0, C		145.3, C		144.9, C		142.5, C		143.3, C	
4	106.9, CH	6.88, s	107.3, CH	6.87, s	106.9, CH	6.89, s	106.0, CH	6.70, s	106.3, CH	6.71, s
4a	155.0, C		155.5, C		155.0, C		154.9, C		155.1, C	
5	105.3 C		105.0, C		104.8, C		107.8, C		107.1, C	
6	120.6, CH	7.69, s	121.0, CH	7.69, s	120.7, CH	7.73, s	122.0, CH	7.31, s	122.7, CH	
7	119.6, CH		119.2, C		118.9, C		124.7, C		122.3, C	
7a	111.1, C		111.4, C		111.1, C		110.7, C		110.9, C	
8	176.2, C		176.6, C		176.2, C		174.7, C		175.7, C	
8a	107.7, C		108.1, C		107.6, C		107.3, C		107.4, C	
9										
9a	160.6, C		160.7, C		160.3, C		154.2, C		155.0, C	
10	21.7, CH_3_	2.38, s	21.7, CH_3_	2.38, s	21.7, CH_3_	2.38, s	21.5, CH_3_	2.32, s	21.6, CH_3_	2.32, s
11	145.1, CH	8.74, d (14.5) ^a^	146.1, C	8.68, d, (14.5) ^a^	145.2, CH	8.73, d (14.7) ^a^	147.0, CH	8.50, s	152.1, CH	8.61, s
12	164.2, C		164.7, C		164.2, C		164.9, C		165.0, C	
13	51.2, CH_3_	3.77, s	51.6, CH_3_	3.77, s	51.1, CH_3_	3.76, s	50.0, CH_3_	3.65, s	50.3, CH_3_	3.76, s
14					55.5, CH_3_	3.84, s	55.1, CH_3_	3.75, s		
1′	140.7, C (10.3) ^b^		135.5, C (2.6) ^b^		140.1, C		133.1, C		133.7, C *	
2′	105.28, CH (26.2) ^b^	7.61, d (10.7) ^c^	120.5, CH, (8.3) ^b^	7.68, m	104.0, CH	7.23, t (2.0) ^a^	121.4, CH	7.10, d (8.7) ^a^	112.7, CH *	7.10, brd
3′	162.8, C (244.9) ^b^		116.5, CH, (23.0) ^b^	7.36, t (8.7) ^a^	160.4, C		114.2, CH	6.91, d (8.8) ^a^	125.5, CH *	7.49, m *
4′	112.6, CH (21.4) ^b^	7.12, t (8.0) ^c^	160.6, C (244.8) ^b^		111.9, CH	6.87, dd (8.0, 2.0) ^a^	156.1, C		123.3, CH	7.65, d (7.3) ^a^
4a′									134.2, C	
5′	131.5, CH (9.4) ^b^	7.53, q (8.0) ^a^			130.7, CH	7.40, t (8.1) ^a^			127.8, CH	7.88, d (7.0) ^a^
6′	114.8, CH (2.8) ^b^	7.44, d (8.0) ^a^			110.7, CH	7.21, dd (8.1, 2.0) ^a^			123.6, CH *	7.49, m *
7′									126.7, CH *	7.49, m *
8′									124.0, CH	8.39, d (7.0) ^a^
8a′									129.0, C	
OH		13.75, brd		13.84, brd		13.83, brd		14.97, brd		14.91, brd
NH		11.84, d (14.5) ^a^		11.89, d (14.5) ^a^		11.83, d (14.7) ^a^				

## Data Availability

NMR and MS raw files can be requested from the authors.

## Supplementary Materials

The following are available online. NMR spectra of compounds **1**–**5** (S1–S32), HPLC chromatograms (S33 and S34), vorinostats made in this study and scope of biotransformation (S35 and S36)

## Sample Availability

Samples of the compounds, if available in enough quantity, are available from the authors.
