# Supplementary material for: Precursor-Directed Biosynthesis of Aminofulvenes: New Chalanilines from Endophytic Fungus Chalara sp
Source: Molecules. 2021 Jul 22;26(15):4418. doi: 10.3390/molecules26154418 (PMC8347292; doi:10.3390/molecules26154418)
Supplement: Supplementary file 1 [file molecules-26-04418-s001.zip › molecules-1283039-supplementary.pdf]

Supplemental Information

Precursor-directed Biosynthesis of Aminofulvenes: new Chalanilines from endophytic fungus *Chalara* sp.

Mahsa Khoshbakht [1], Jason Srey [1], Donovon A. Adpressa [1], Annika Jagels [2], Sandra Loesgen [1,2,*]

[1] Department of Chemistry, Oregon State University, Corvallis, Oregon 97331, United States

[2] Present Address: Whitney Laboratory for Marine Bioscience, Department of Chemistry, University of Florida, St. Augustine, Florida 32080, United States.

* Address for correspondence

-Table of content-

**Figure S1**	^{1}H NMR spectrum of **1**	
**Figure S2**	Expanded ^{1}H NMR spectrum of **1**	
**Figure S3**	^{13}C NMR spectrum of **1**	
**Figure S4**	Expanded ^{13}C NMR spectrum of **1**	
**Figure S5**	COSY NMR spectrum of **1**	
**Figure S6**	HSQC NMR spectrum of **1**	
**Figure S7**	HMBC NMR spectrum of **1**	
**Figure S8**	^{1}H NMR spectrum of **2**	
**Figure S9**	Expanded ^{1}H NMR spectrum of **2**	
**Figure S10**	^{13}C NMR spectrum of **2**	
**Figure S11**	Expanded ^{13}C NMR spectrum of **2**	
**Figure S12**	COSY NMR spectrum of **2**	
**Figure S13**	HSQC NMR spectrum of **2**	
**Figure S14**	HMBC NMR spectrum of **2**	
**Figure S15**	^{1}H NMR spectrum of **3**	
**Figure S16**	Expanded ^{1}H NMR spectrum of **3**	
**Figure S17**	^{13}C NMR spectrum of **3**	
**Figure S18**	COSY NMR spectrum of **3**	
**Figure S19**	HSQC NMR spectrum of **3**	
**Figure S20**	HMBC NMR spectrum of **3**	
**Figure S21**	^{1}H NMR spectrum of **4**	
**Figure S22**	Expanded ^{1}H NMR spectrum of **4**	
**Figure S23**	^{13}C NMR spectrum of **4**	

**Figure S24**	COSY NMR spectrum of **4**	
**Figure S25**	HSQC NMR spectrum of **4**	
**Figure S26**	HMBC NMR spectrum of **4**	
**Figure S27**	^{1}H NMR spectrum of **5**	
**Figure S28**	Expanded ^{1}H NMR spectrum of **5**	
**Figure S29**	^{13}C NMR spectrum of **5**	
**Figure S30**	COSY NMR spectrum of **5**	
**Figure S31**	HSQC NMR spectrum of **5**	
**Figure S32**	HMBC NMR spectrum of **5**	
**Figure S33**	Biotransformation of vorinostat analogs into chalaniline A type molecules	
**Figure S34**	Time course of chalaniline A production	
**Figure S35**	Presence of chalanilines in culture broth, not fungal cells	
**Figure S36**	Vorinostat derivatives made in this study	

*All the experiments and their NMR data were collected at room temperature.

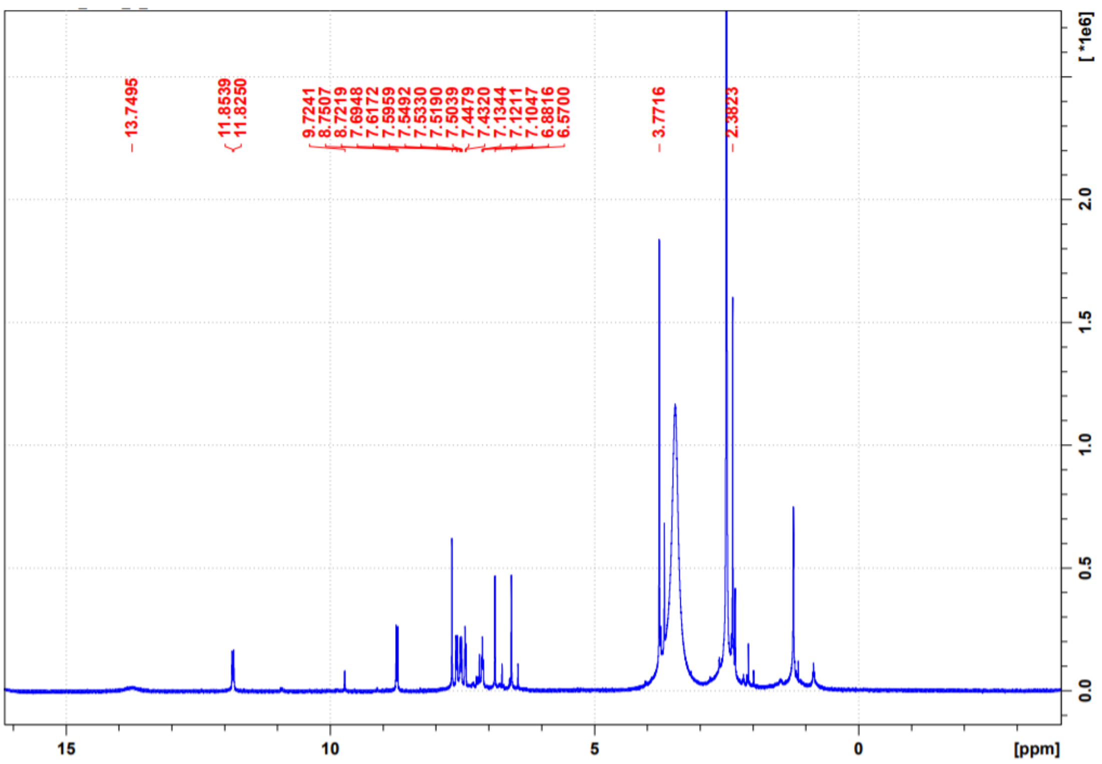

Figure S1. ^{1}H NMR spectrum (500 MHz, DMSO) of **1**.

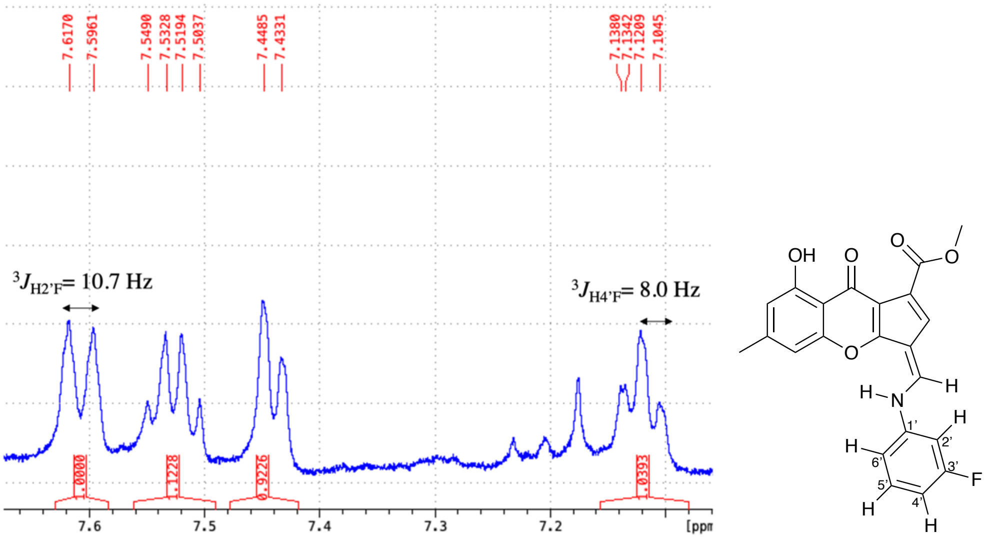

Figure S2. Expanded ^{1}H NMR spectrum of the H-F couplings of **1**.

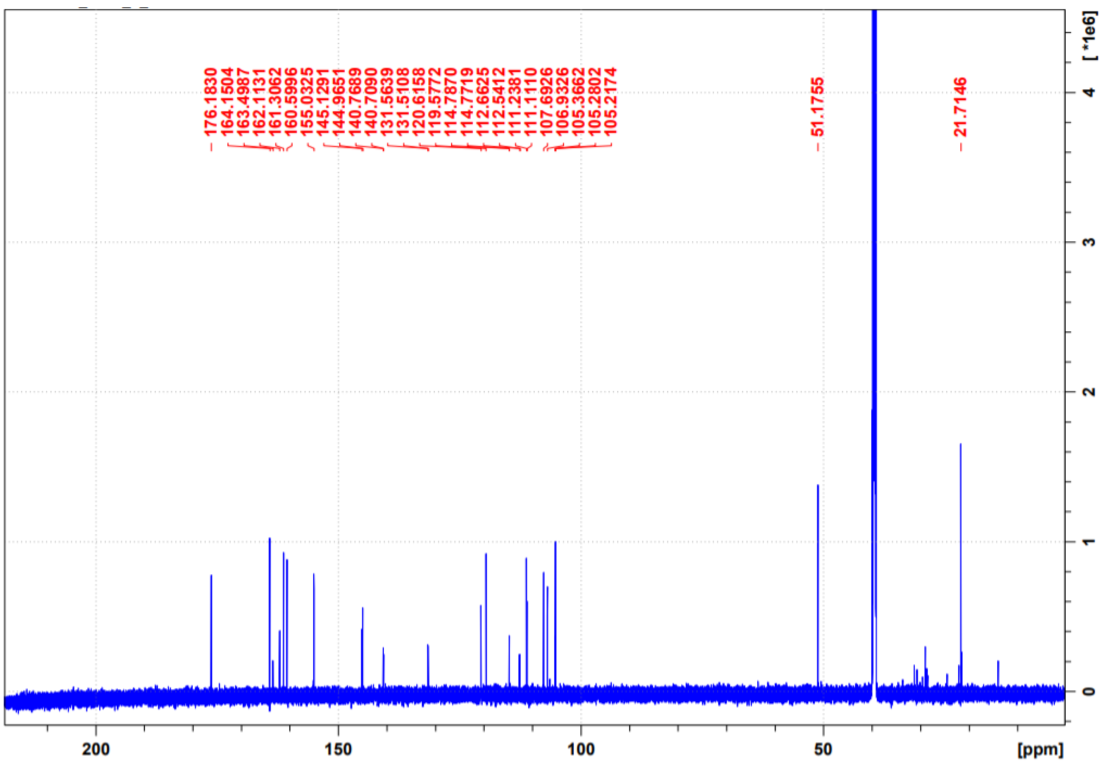

Figure S3. ^{13}C NMR spectrum (176 MHz, DMSO) of **1**.

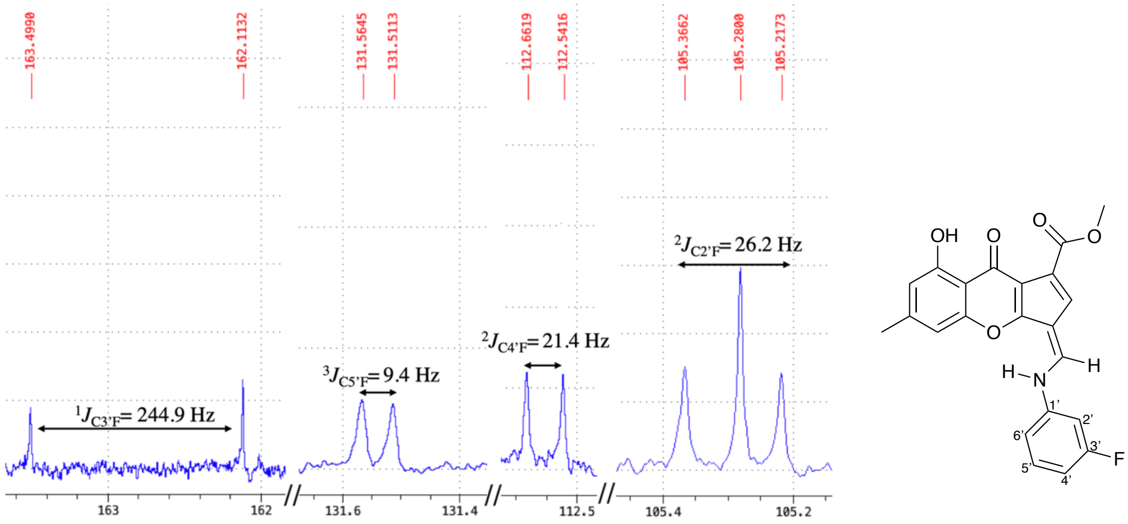

Figure S4. Expanded ^{13}C NMR spectrum of the C-F couplings of **1**.

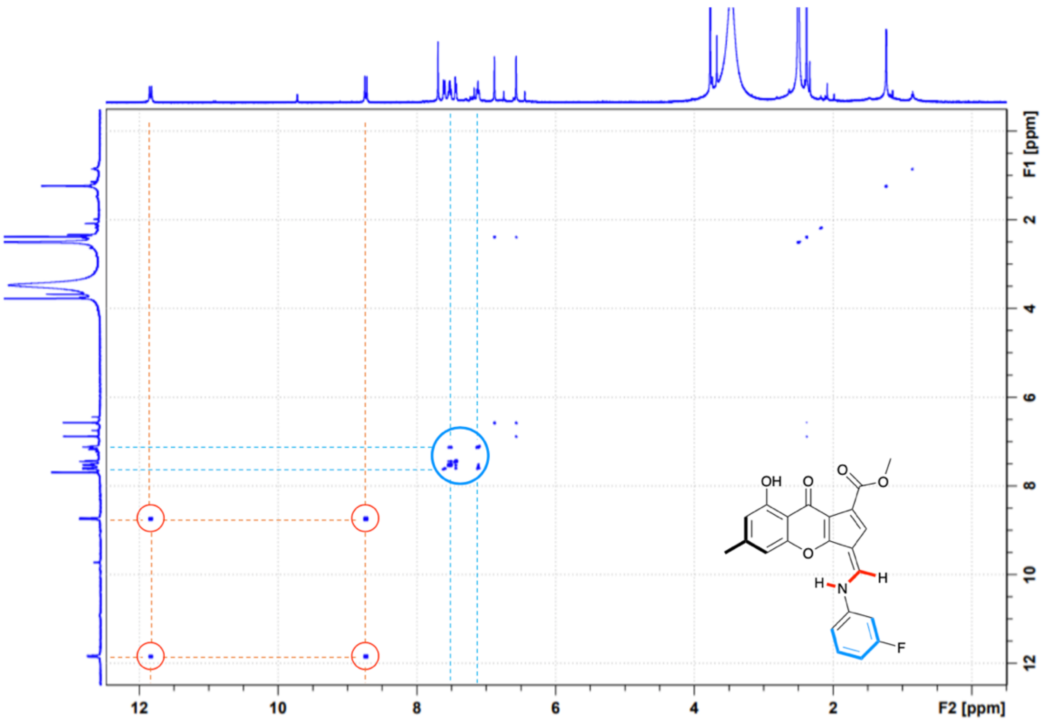

Figure S5. COSY NMR spectrum of **1**.

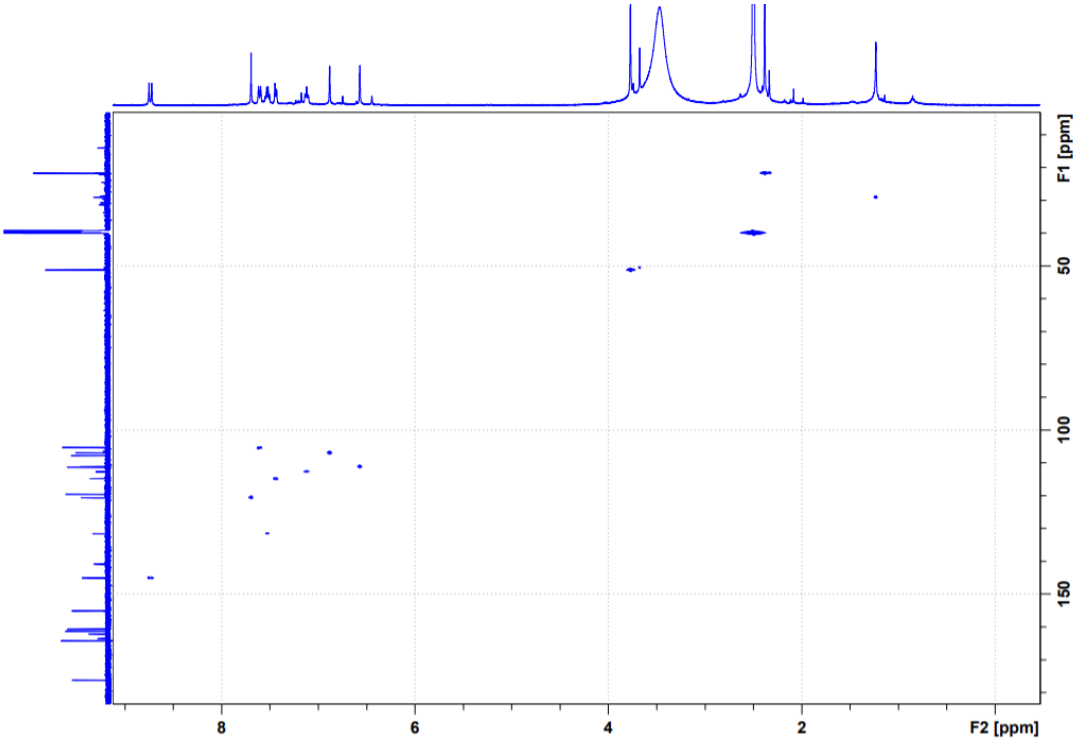

Figure S6. HSQC NMR spectrum of **1**.

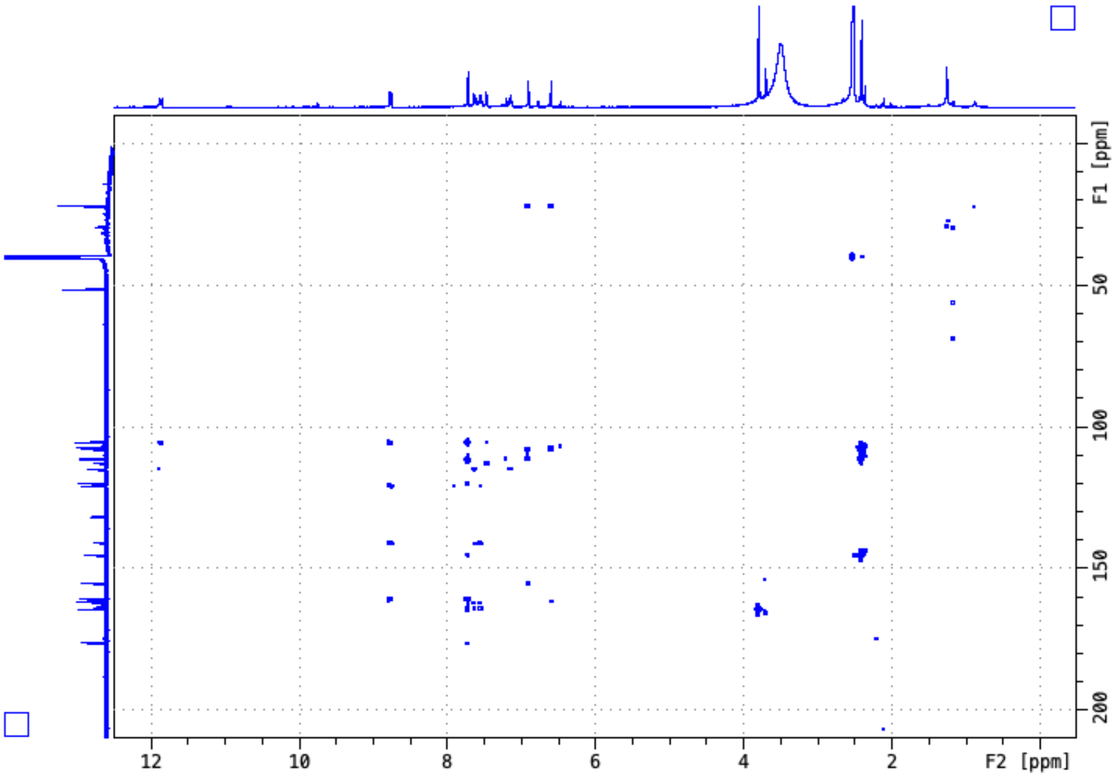

Figure S7. ^{1}H-^{13}C HMBC NMR spectrum of **1**.

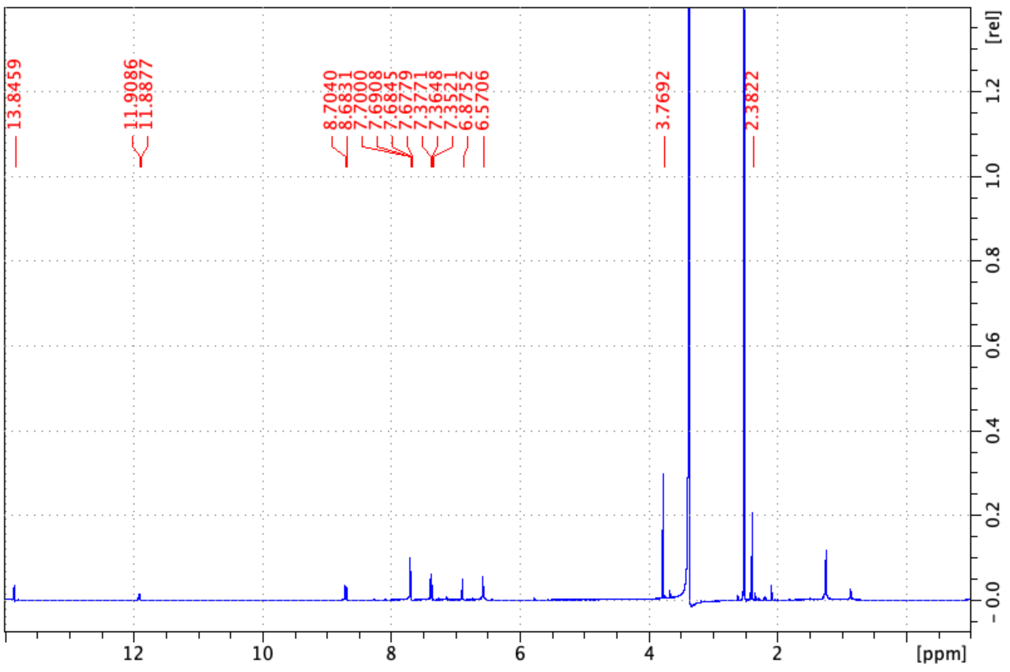

Figure S8. ^{1}H NMR spectrum (700 MHz, DMSO) of **2**.

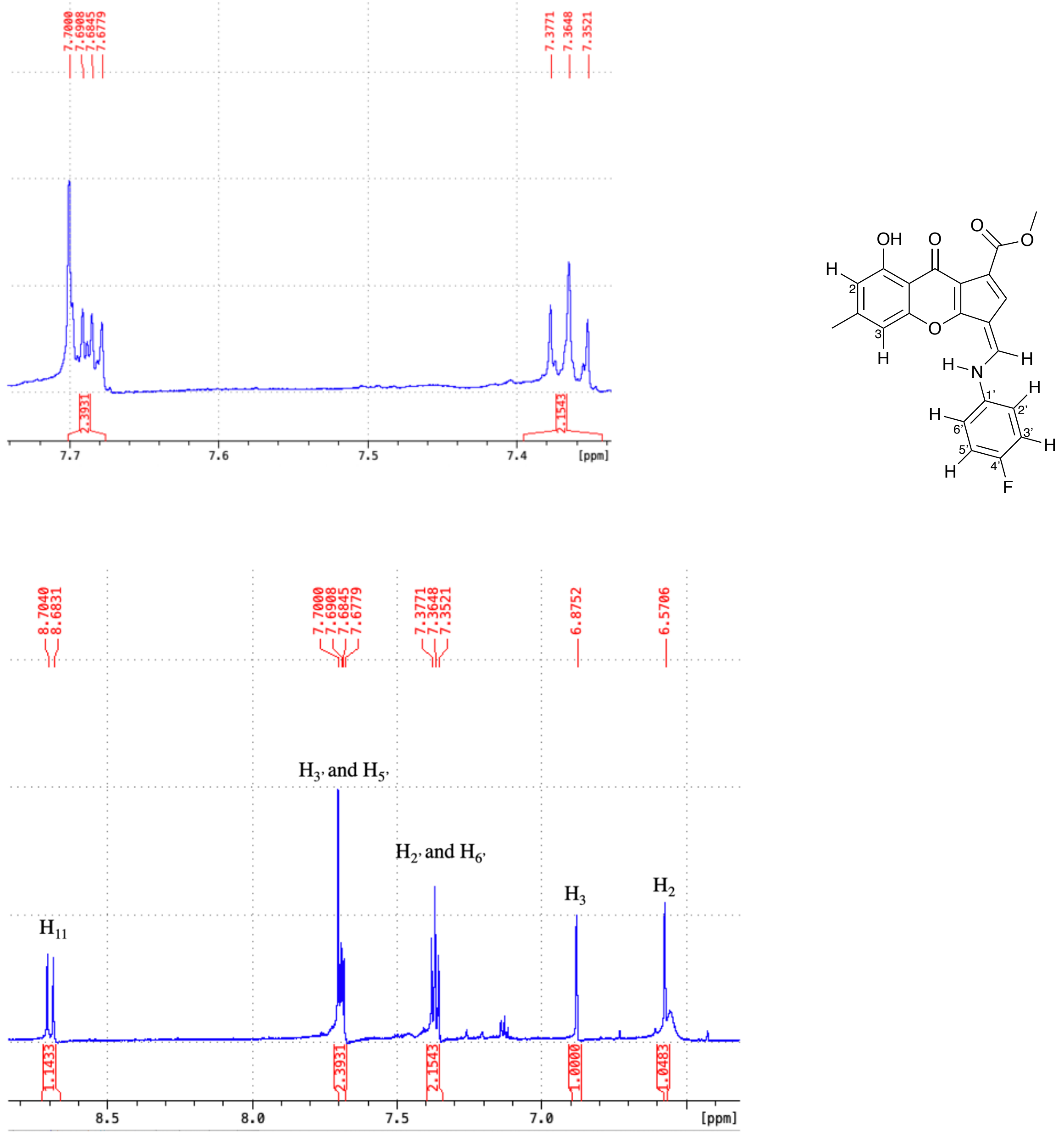

Figure S9. Expanded ^{1}H NMR spectrum of the H-F couplings of **2**.

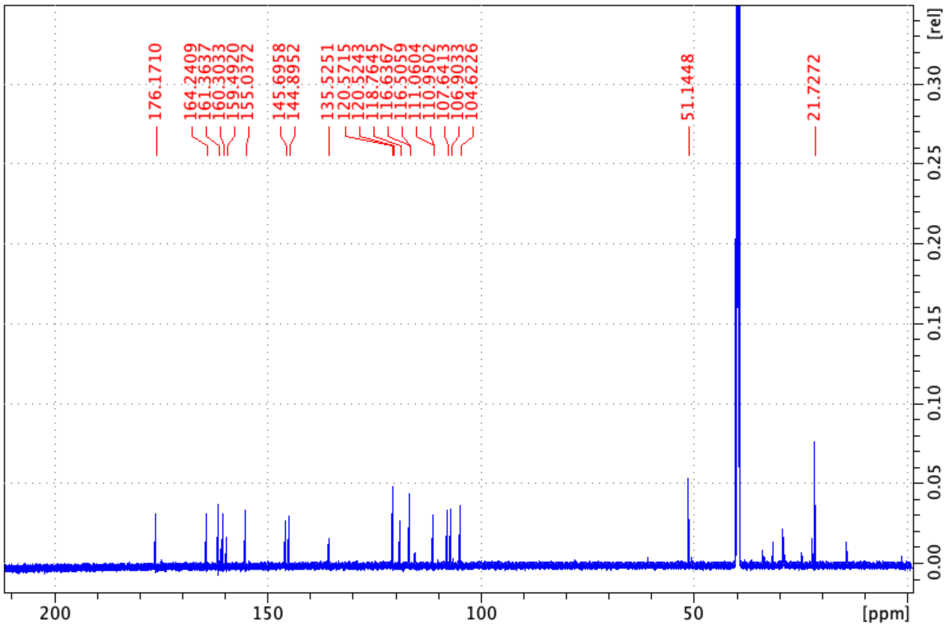

Figure S10. ^{13}CNMR spectrum (176 MHz, DMSO) of **2**.

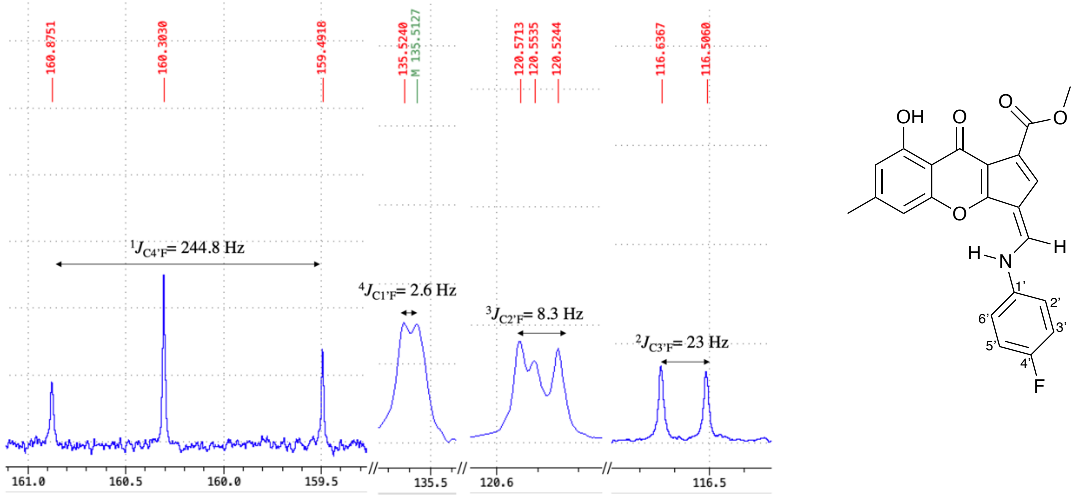

Figure S11. Expanded ^{13}C NMR spectrum of the C-F couplings of **2**.

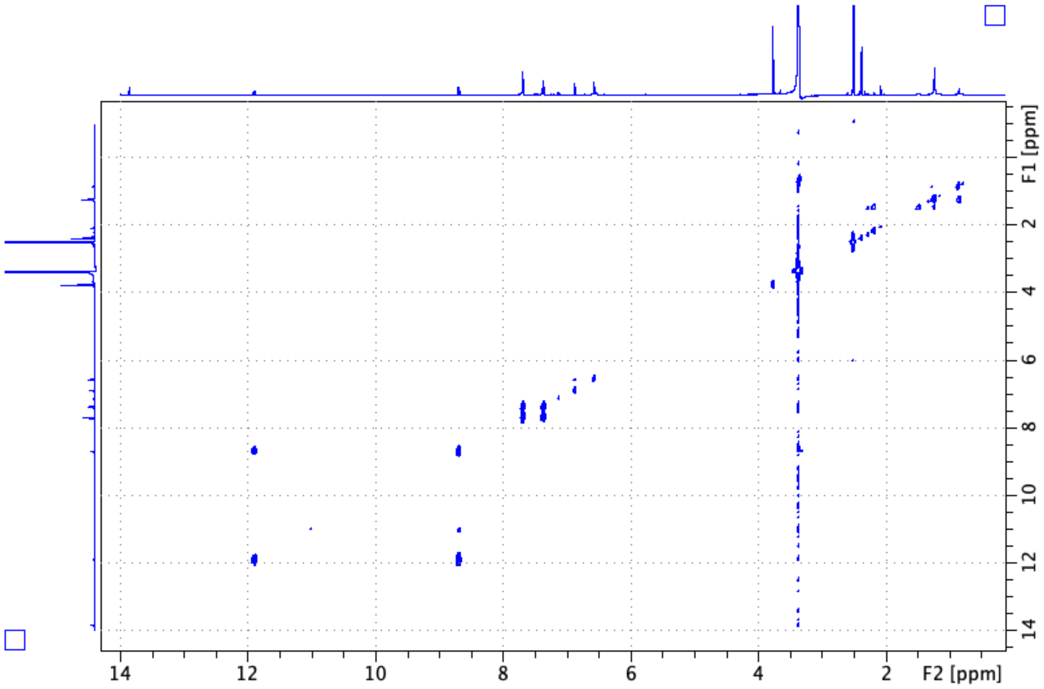

Figure S12. COSY NMR spectrum of **2**.

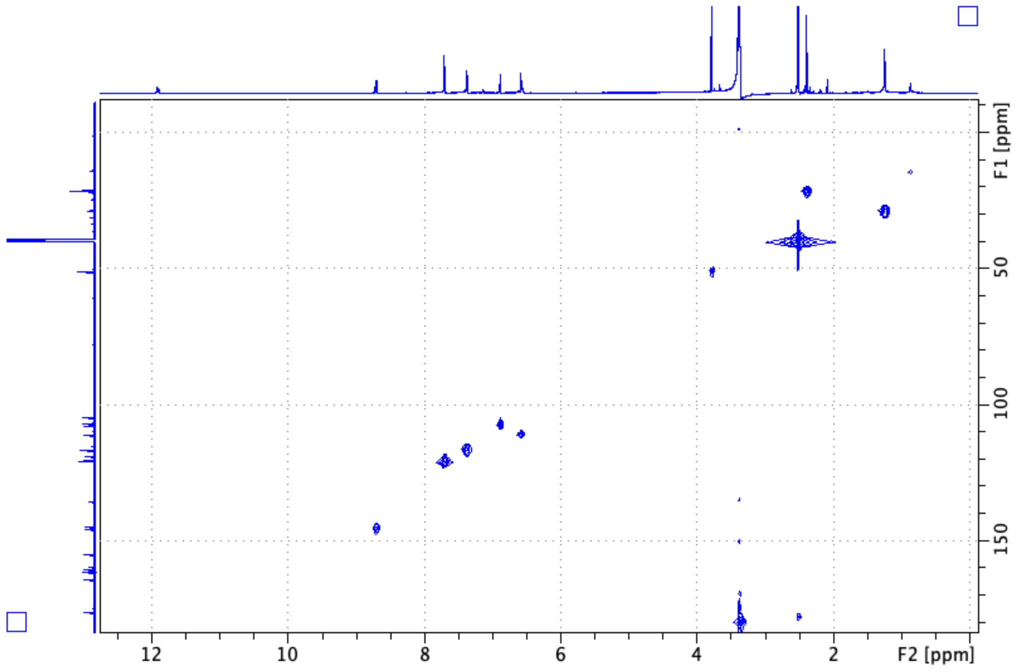

Figure S13. HSQC NMR spectrum of **2.**

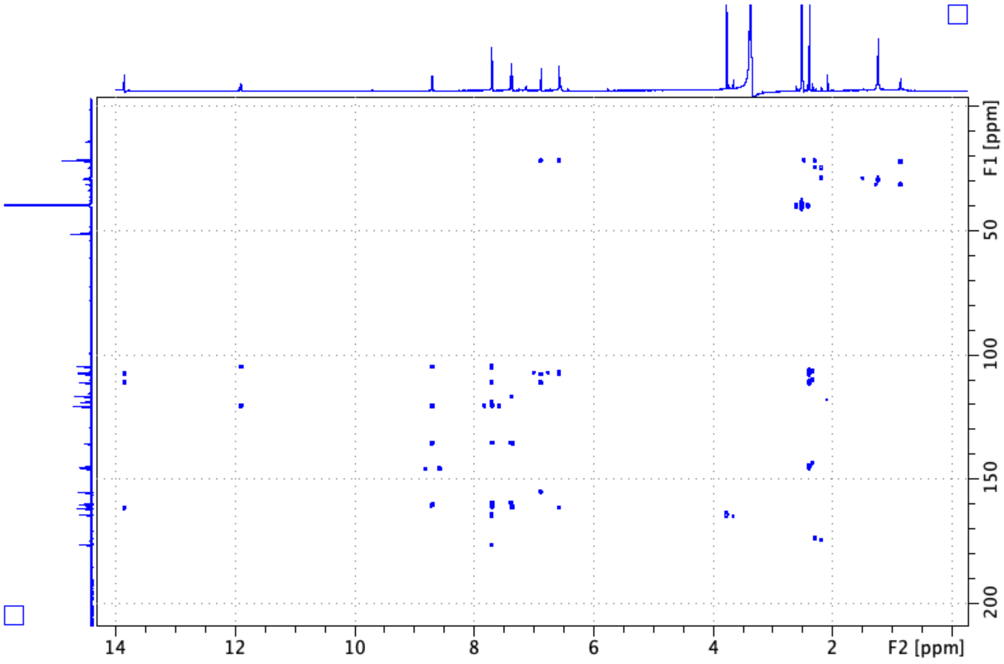

Figure S14. ^{1}H-^{13}C HMBC NMR spectrum of **2**.

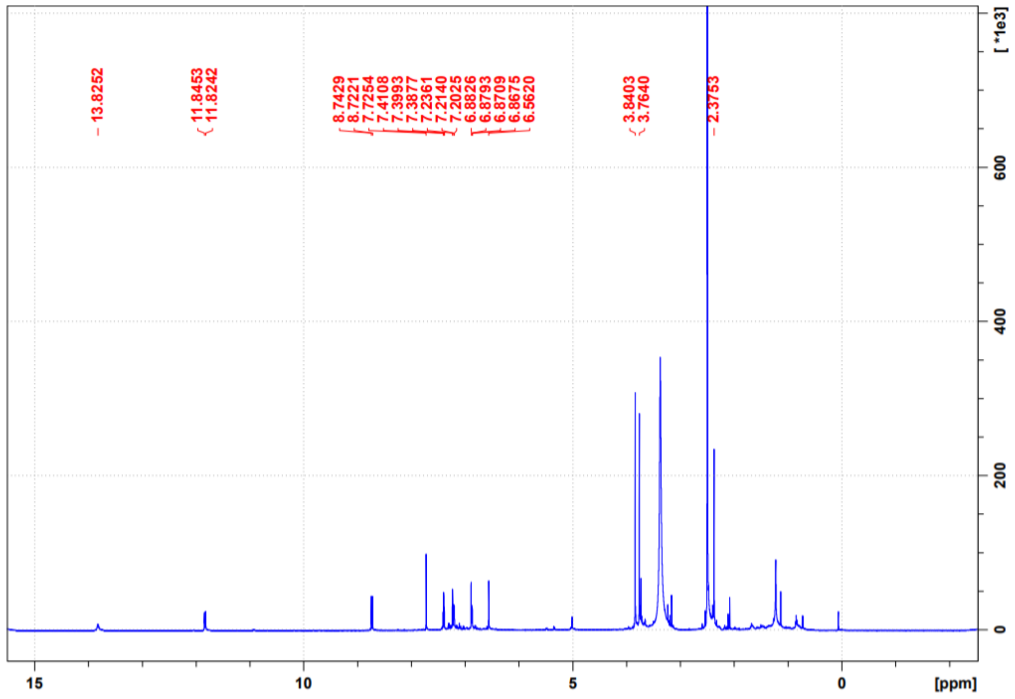

Figure S15. ^{1}H NMR spectrum (700 MHz, DMSO) of **3**.

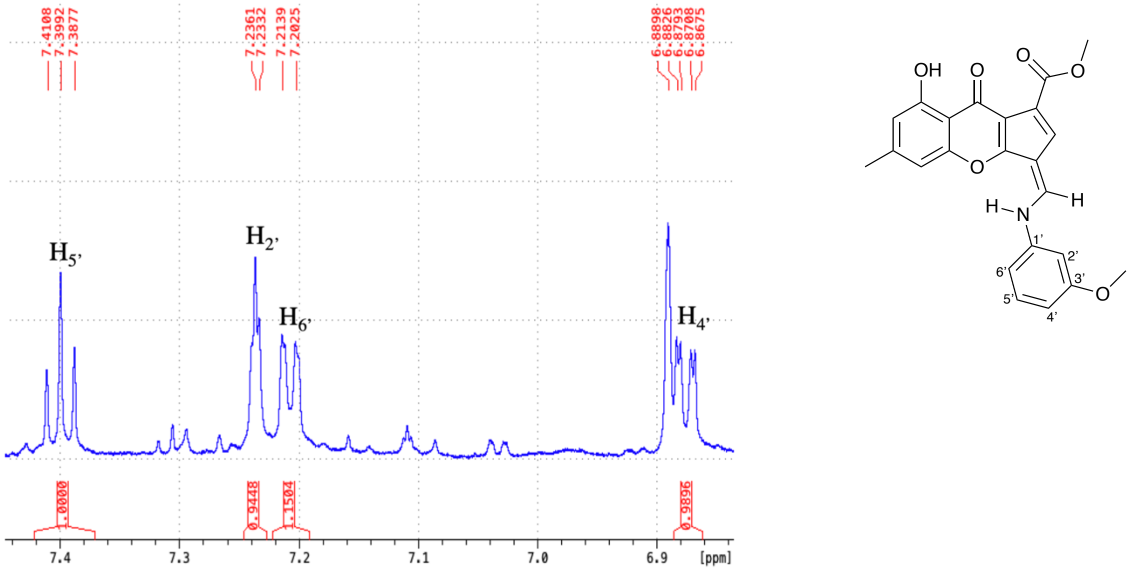

Figure S16. Expanded aromatic region of the ^{1}H NMR spectrum of **3**.

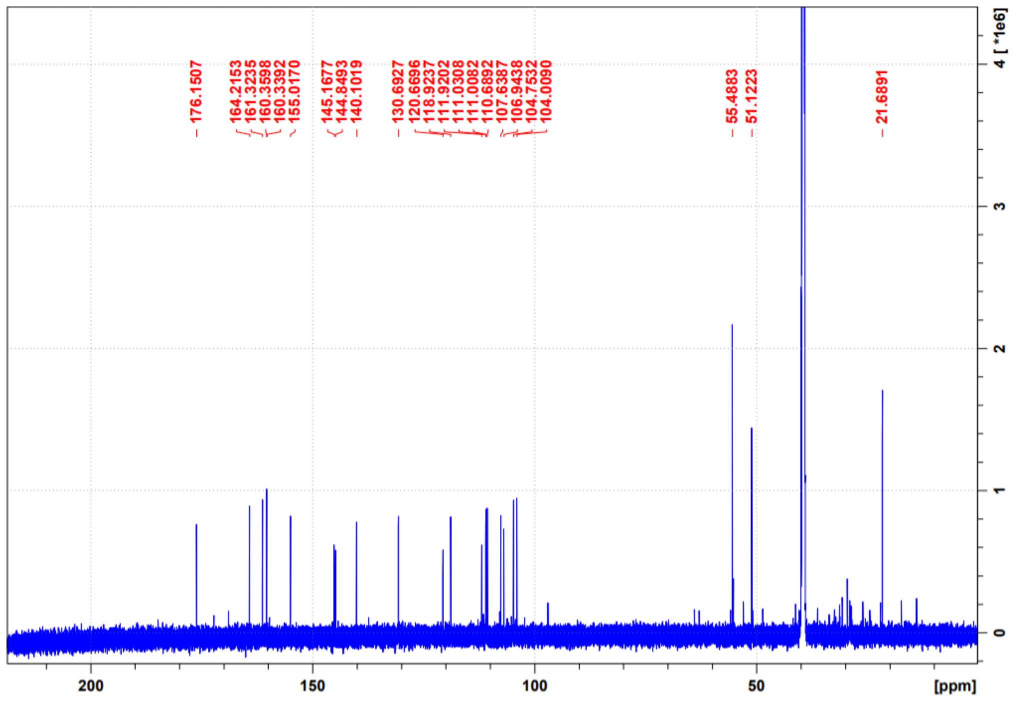

Figure S17. ^{13}C NMR spectrum (176 MHz, DMSO) of **3**.

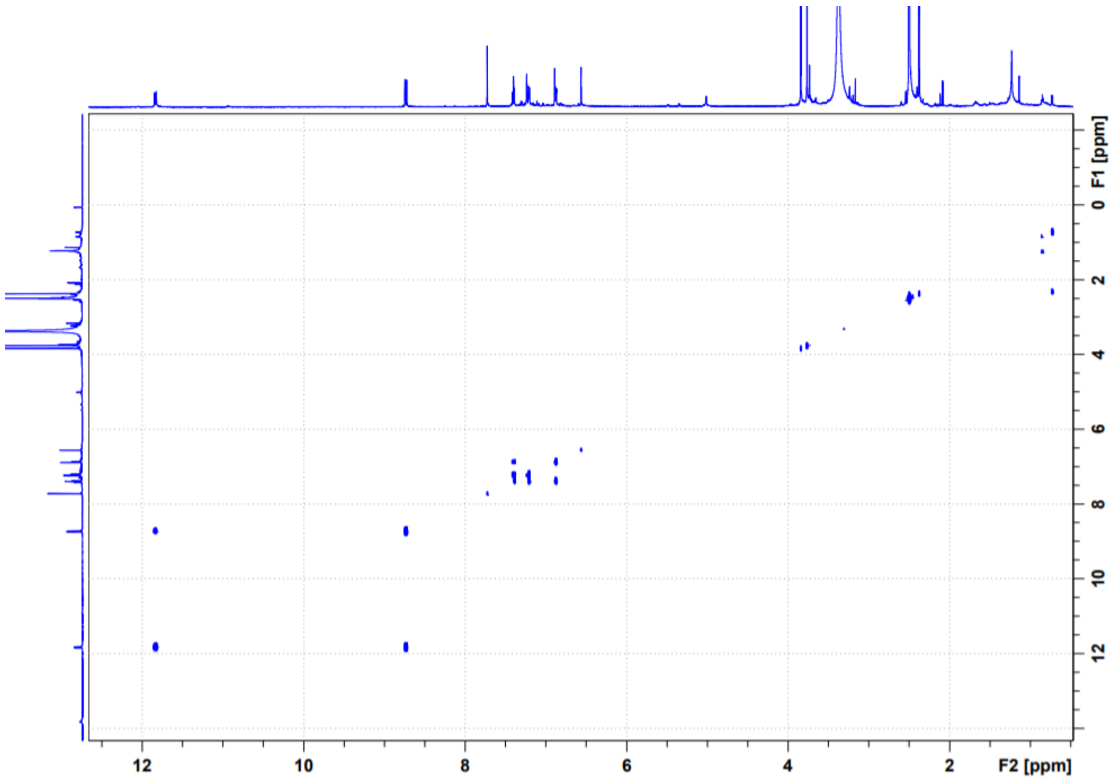

Figure S18. COSY NMR spectrum of **3**.

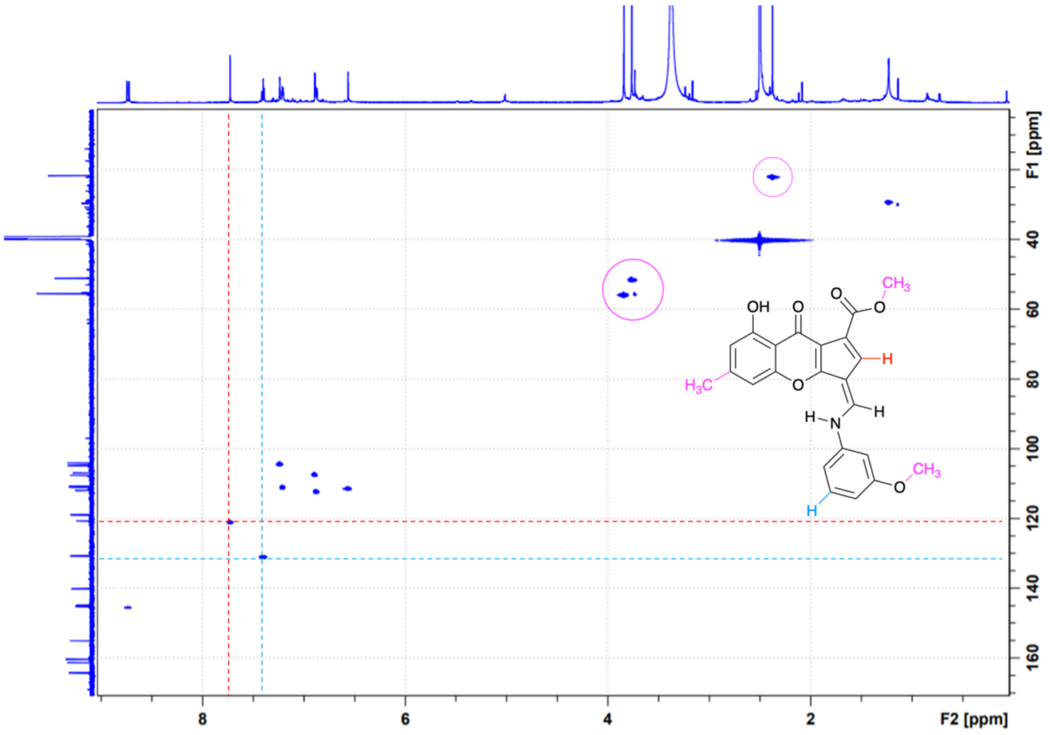

Figure S19. HSQC NMR spectrum of **3**.

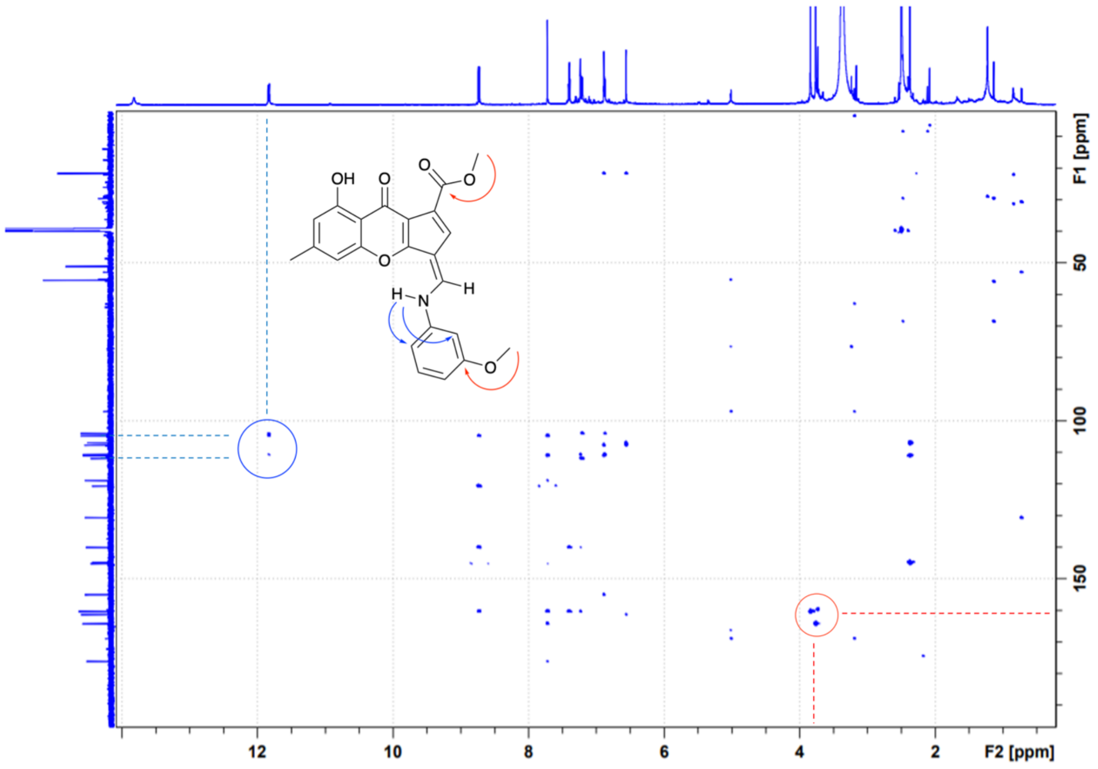

Figure S20. ^{1}H-^{13}C HMBC NMR spectrum of **3**.

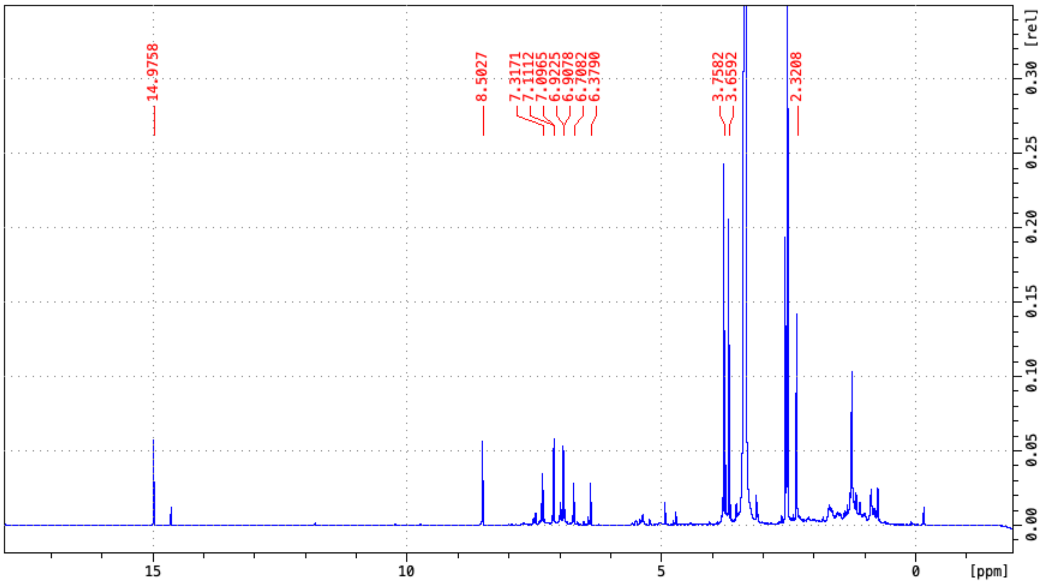

Figure S21. ^{1}H NMR spectrum (600 MHz, DMSO) of **4**.

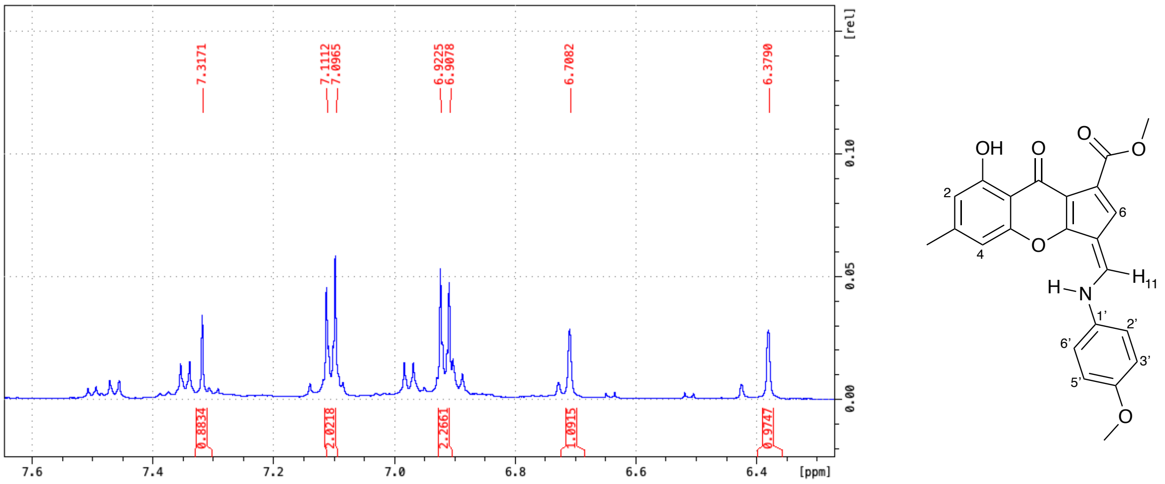

Figure S22. Expanded aromatic region of the ^{1}H NMR spectrum of **4**.

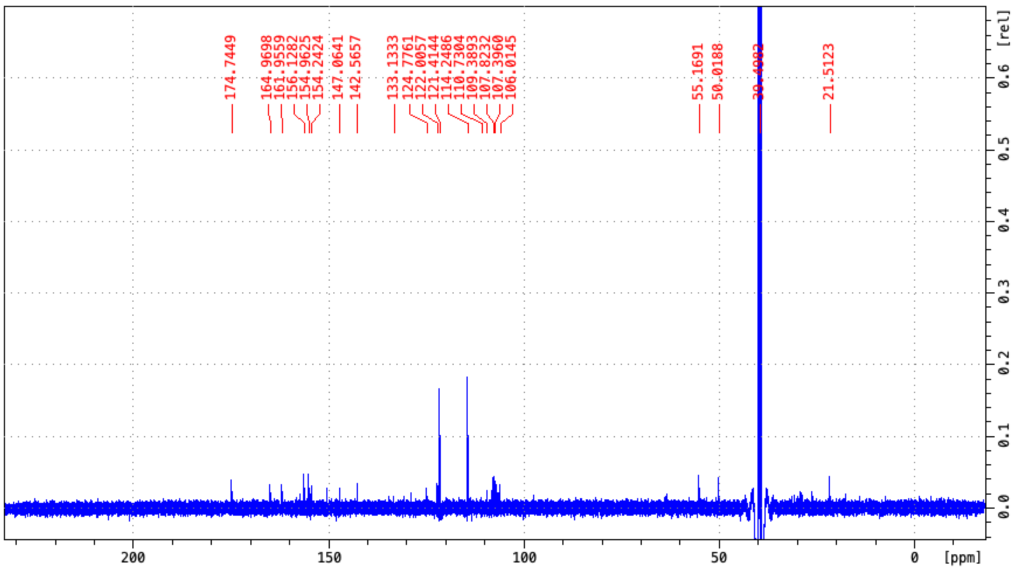

Figure S23. ^{13}C NMR spectrum (150 MHz, DMSO) of **4**.

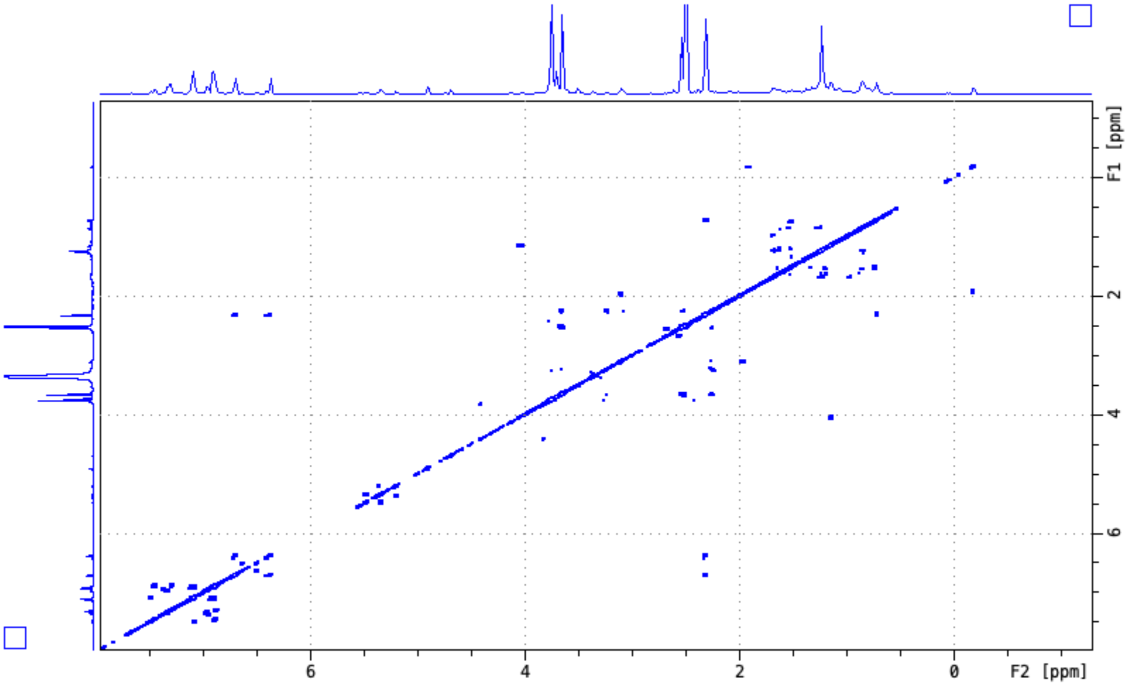

Figure S24. COSY NMR spectrum of **4**.

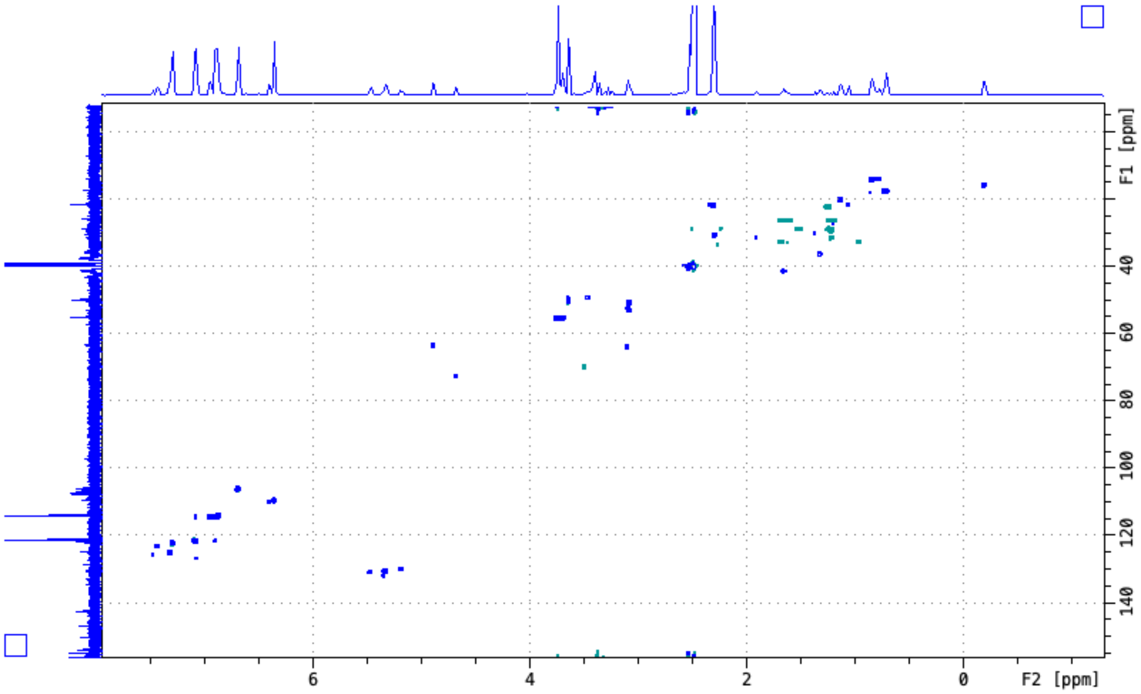

Figure S25. HSQC NMR spectrum of **4**.

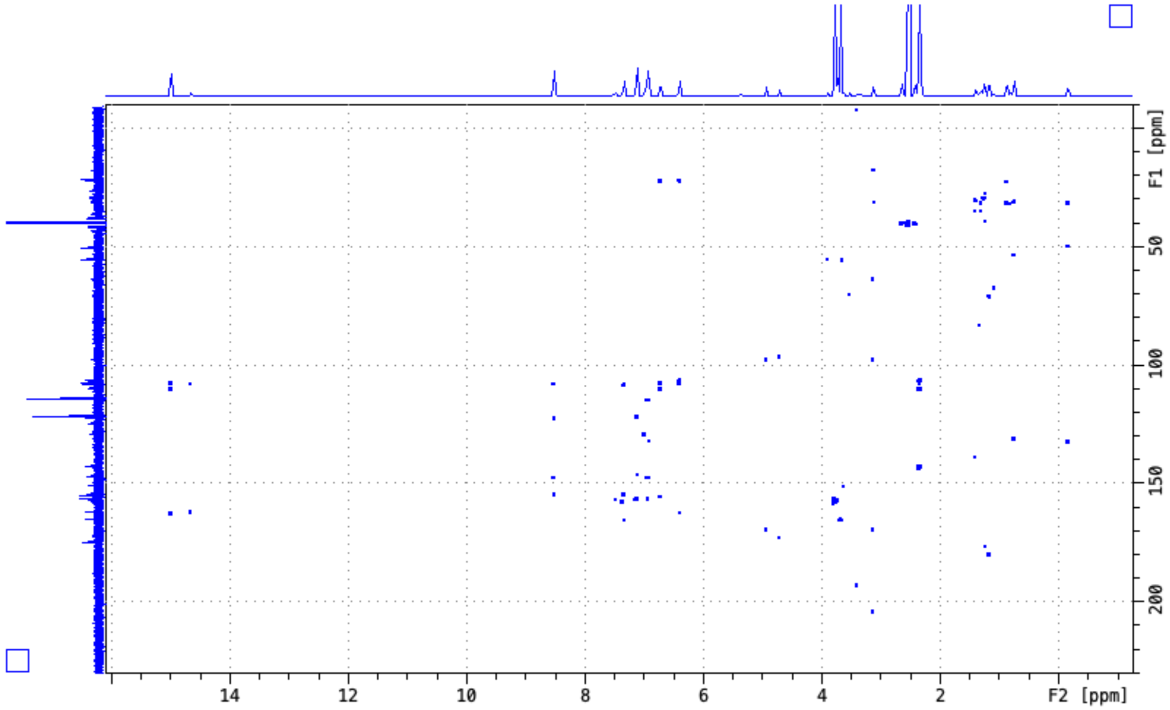

Figure S26. ^{1}H-^{13}C HMBC NMR spectrum of **4**.

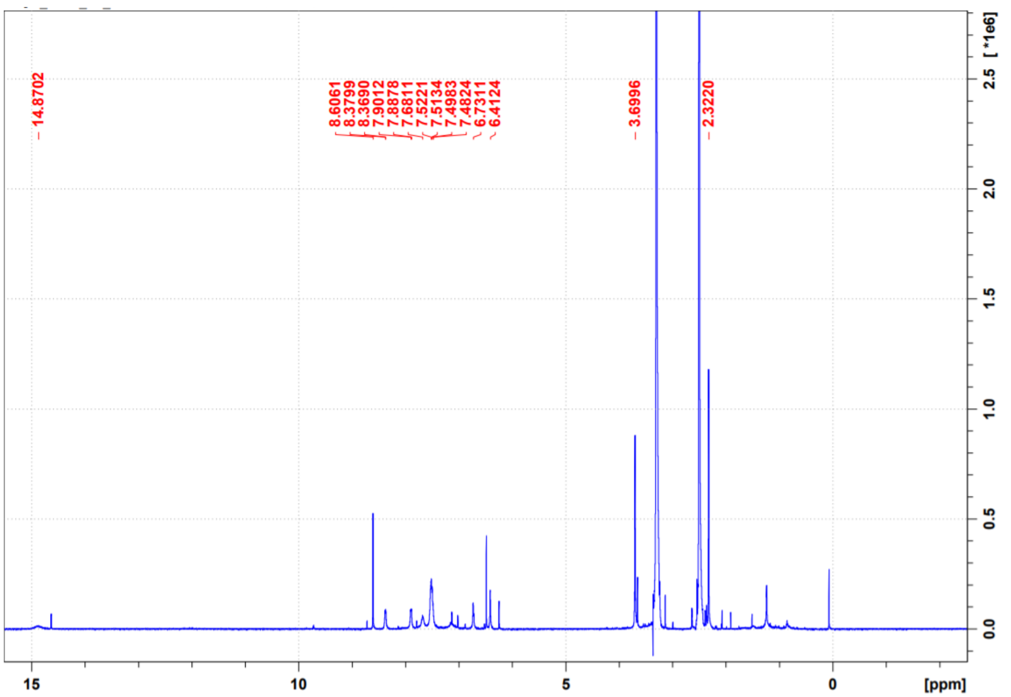

Figure S27. ^{1}H NMR spectrum (500 MHz, DMSO) of **5**.

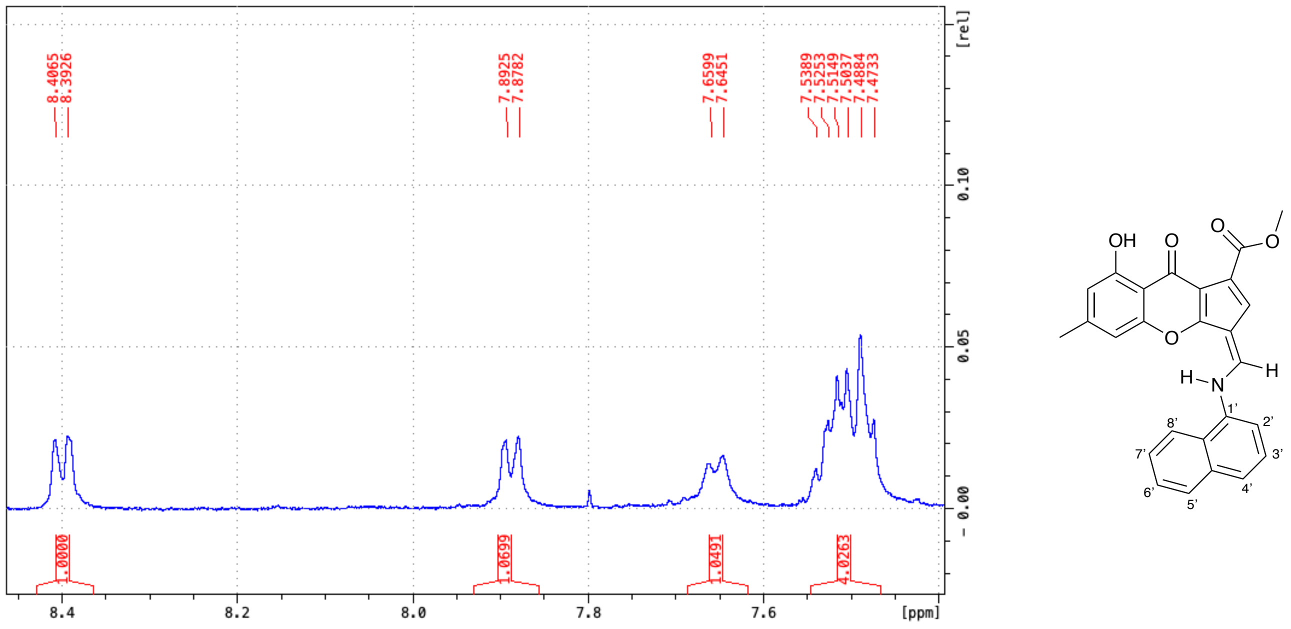

Figure S28. Expanded ^{1}H NMR spectrum of the naphthyl group of **5**.

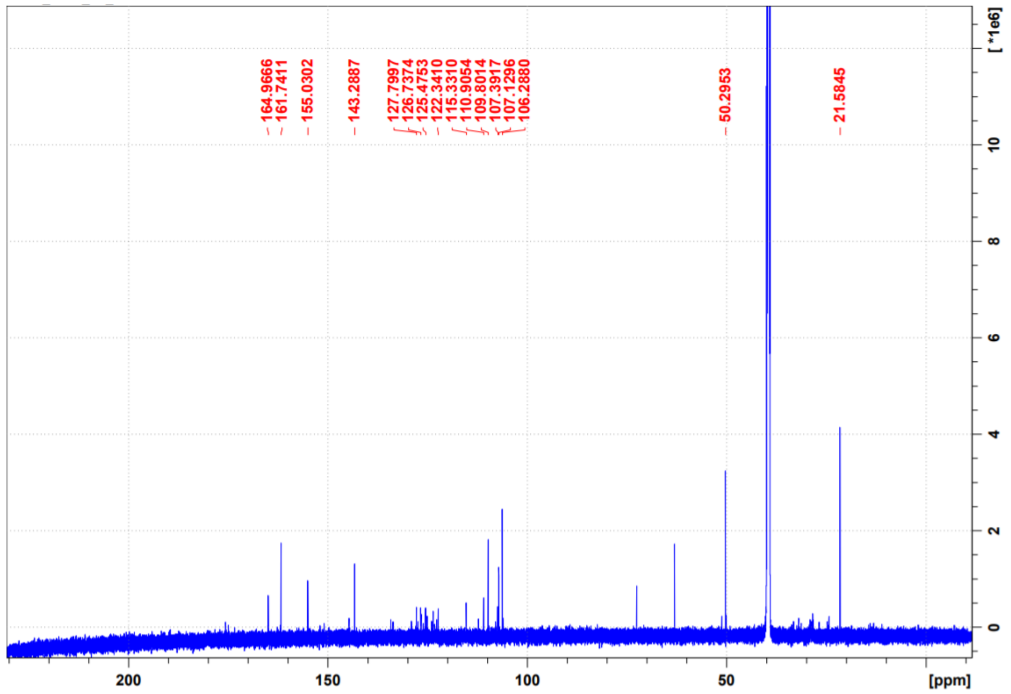

Figure S29. ^{13}C NMR spectrum (176 MHz, DMSO) of **5**.

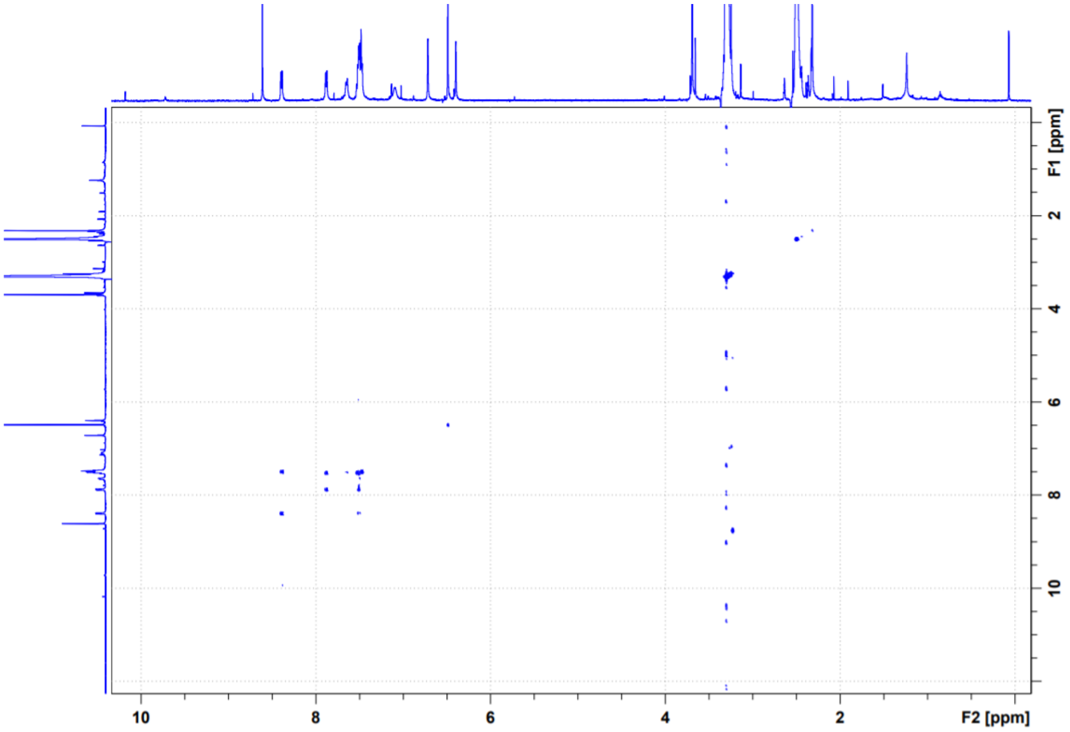

Figure S30. COSY NMR spectrum of **5.**

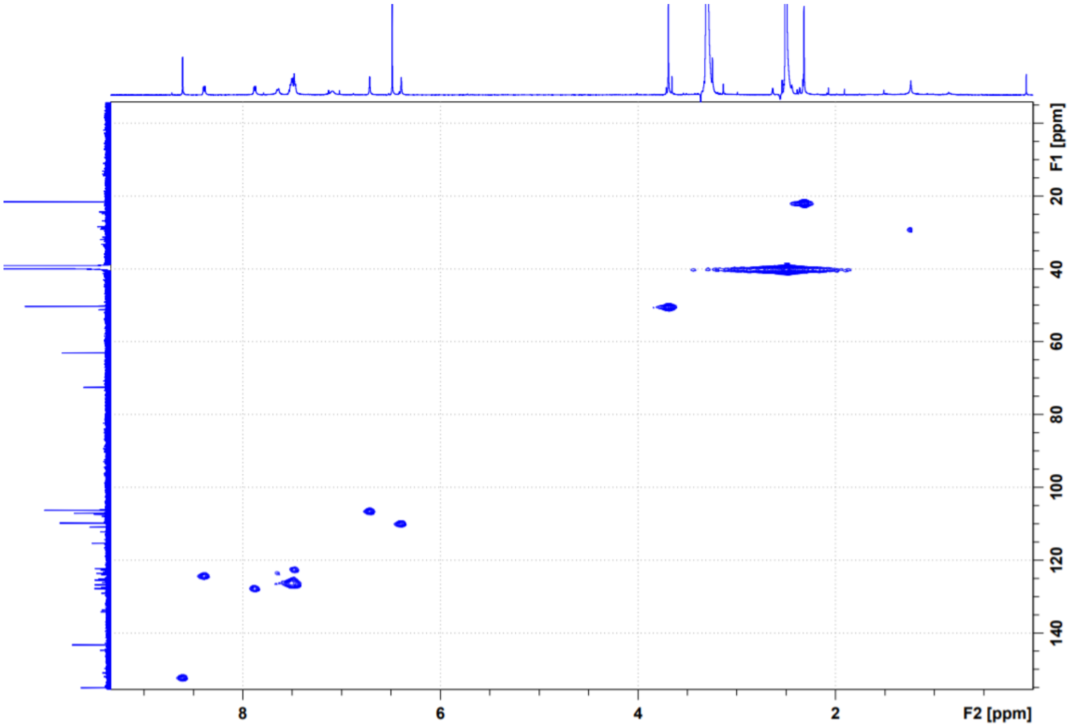

Figure S31. HSQC NMR spectrum of **5**.

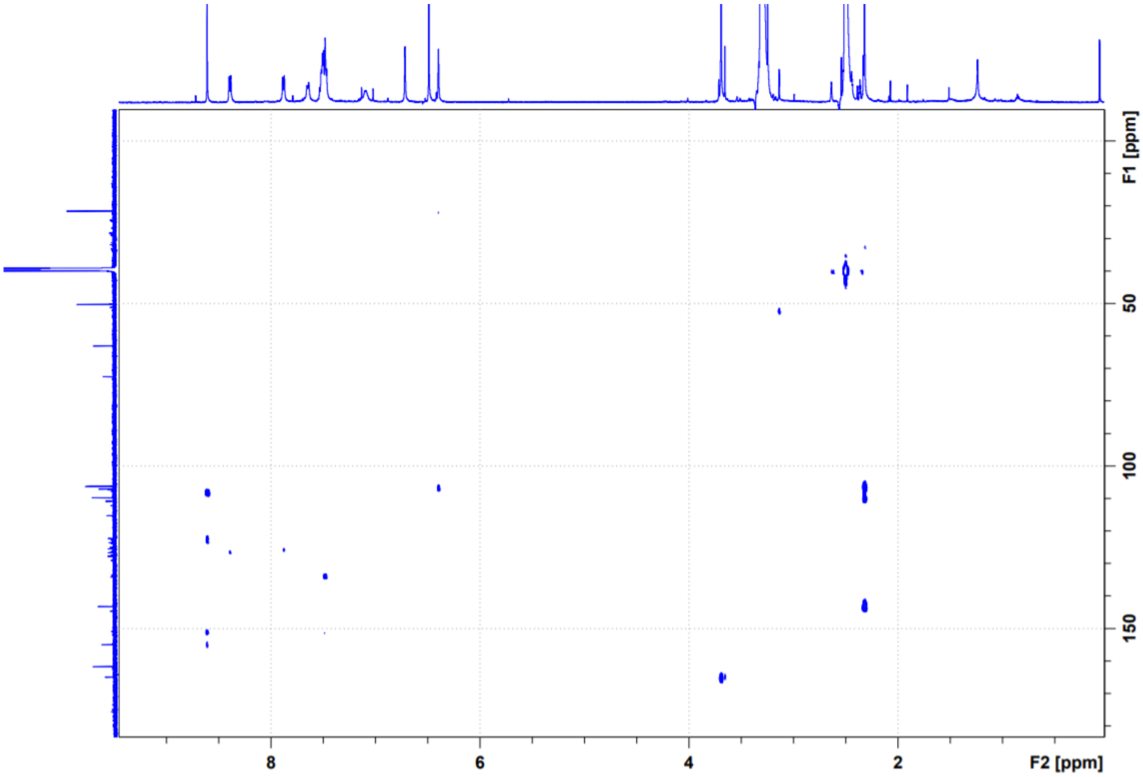

Figure S32. ^{1}H-^{13}C HMBC NMR spectrum of **5**.

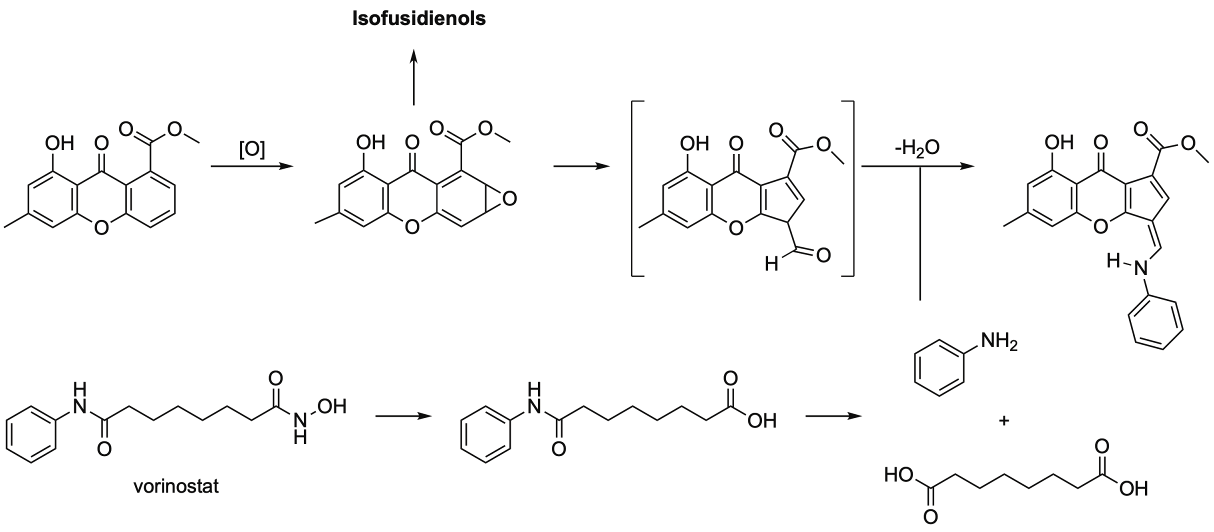

Figure S33. Biosynthetic proposal of the formation of chalanilines. Modified from [12].

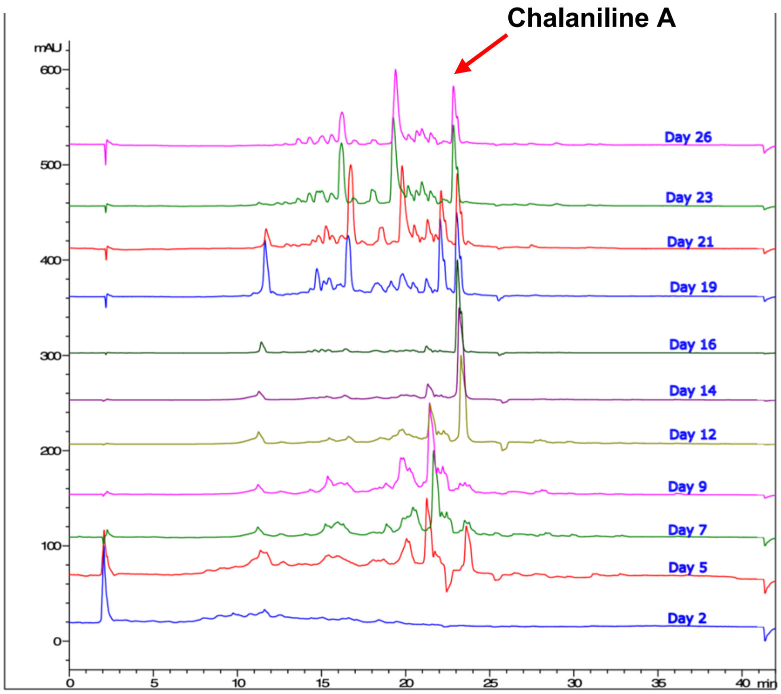

Figure S34. Assessment of chalaniline A (rt 24 min) production from day 2 to 26 of precursor addition with LCMS (UV observed at 360 nm). Highest amount of chalaniline A was observed at day 16.

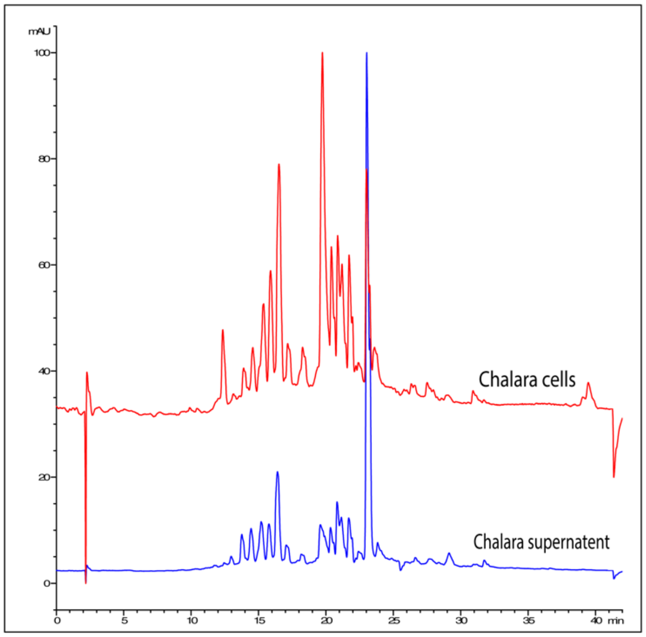

Figure S35. Chalaniline A (rt 24 min) is mainly found in the culture broth (blue) and to a lesser amount in the fungal cells (red).

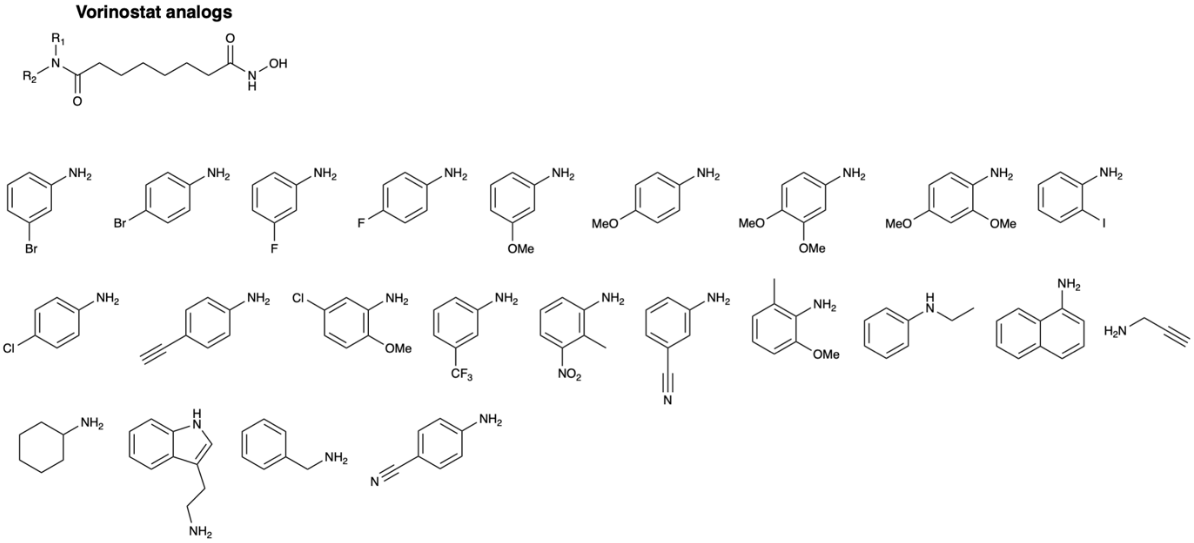

Figure S36. 23 Vorinostat derivatives prepared in this study.

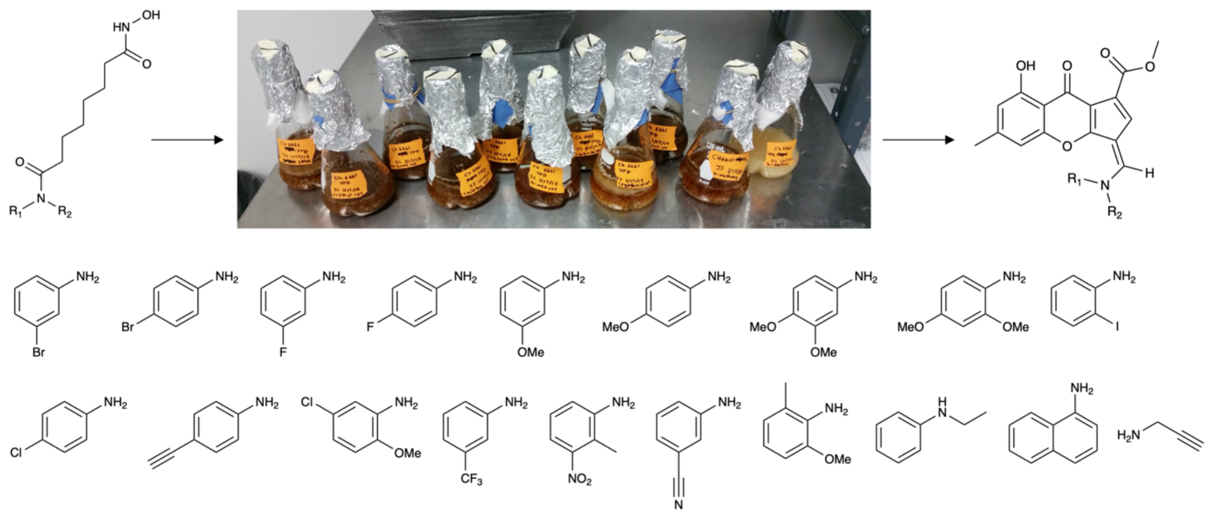

Figure S37. 19 Vorinostat analogs which were successfully incorporated into the chalaniline A structure.
